# A novel fully conjugated COF adorned on 3D-G to boost the “D–π–A” electron regulation in oxygen catalysis performance†

**DOI:** 10.1039/d5sc02082d

**Published:** 2025-04-22

**Authors:** Yinggang Sun, Wenjie Duan, Jigang Wang, Peng Sun, Yanqiong Zhuang, Zhongfang Li

**Affiliations:** a College of Chemistry and Chemical Engineering, Shandong University of Science and Technology Zibo 255000 Shandong P. R. China zhfli@sdut.edu.cn

## Abstract

Covalent organic frameworks (COFs) are promising materials for oxygen catalysis. Here, a novel, highly stable, conjugated two-dimensional poly(benzimidazole porphyrin-cobalt) (PBIPorCo) with a large delocalization energy is synthesized using meso-5,10,15,20-tetra (4-cyano-phenylporphyrin) cobalt (TCNPorCo) and 3,3′-diaminobenzidine (DAB). The decrease in energy between the HOMO and LUMO orbitals of PBIPorCo could enhance the capability for the gain and loss of electrons during the catalytic process. In a nitrogen-rich environment, a benzimidazole (BI) group can transfer electrons to the Co–N_4_ site and enhance the protonation process in the oxygen reduction reaction (ORR). The π–π interactions between PBIPorCo and three-dimensional graphene (3D-G) form an “electron donor–π–electron acceptor” structure to boost the bifunctional oxygen catalysis process. PBIPorCo/3D-G exhibits outstanding bifunctional oxygen catalytic performance (Δ*E* = 0.62 V) and outstanding performance in zinc–air batteries. It exhibits satisfactory potential for application in fuel cells (FCs) and overall water splitting (OWS). This work presents a promising strategy for the design of novel COFs as bifunctional oxygen catalysts.

## Abstract 2

The BI groups in PBIPorCo transfer electrons to the Co–N_4_ site as a “D–π–A” structure, which enhances the cloud density. The “π–π interactions”.

## Graphical abstract

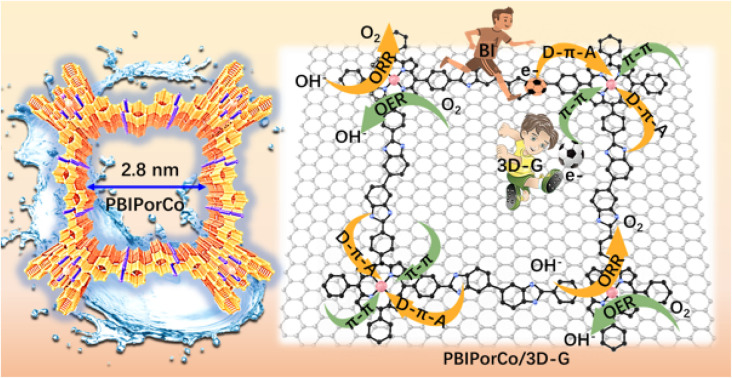

## Introduction

With increasing environmental pollution and depletion of fossil fuel energy sources, there is an urgent need to develop new energy conversion technologies and find alternative clean energy sources.^1,2^ Fuel cells (FCs) are an important class of clean energy conversion devices, where hydrogen oxidation reaction (HOR)^3,4^ occurs at the anode, and oxygen reduction reaction (ORR) occurs at the cathode. Hydrogen is mainly obtained by overall water splitting (OWS), which has a slow kinetic cathode process (oxygen evolution reaction, OER).^5^ OER is the inverse reaction of ORR,^6,7^ so bifunctional oxygen catalysts are significant for FCs and OWS devices. The charge–discharge reactions of metal–air batteries (MABs) correspond to OER and ORR,^8–10^ respectively. The bifunctional oxygen catalysis performance of the catalyst can be exhibited in the charging and discharging process of MABs. Among the various types of MABs, zinc–air batteries (ZABs) are a relatively mature technology that hold great promise for future energy applications. It is essential to achieve high efficiency and design efficient ORR and OER electrocatalysts with high energy conversion.^11,12^ Pt-based materials have been widely used in oxygen catalytic reactions, but high cost and resource limitations hinder their large-scale commercialization. Numerous non-noble metal catalysts have been developed that exhibit more effective electrocatalytic performance than noble metal catalysts.^13,14^ However, the structural changes resulting from heat treatment (HT) often hinder the understanding of the nature of the electrocatalysis.^15–17^

Single-atom catalyst (SAC) strategies have been proposed to enhance the efficiency and utilization of metal atoms in electrocatalysts.^18–20^ In particular, well-distributed M–N_*x*_ sites have the advantage of extremely high metal atom utilization to enhance oxygen catalytic performance.^21,22^ Porphyrin is a natural oxygen catalyst that exists in nature. It is an important raw material for cytochrome P450 for the catalysis of the oxygen reduction process.^13^ Metalloporphyrin (PorM) is a typical SAC, which is often used as a standard sample for synchrotron radiation studies.^23,24^ However, PorM usually decomposes to form metal atomic nanoclusters (M-NCs) during HT, resulting in low metal atom utilization, unclear active sites, and poor stability, so it is important to protect PorM from decomposing during HT. If PorM units were linked to form a fully conjugated polymer, the delocalization energy would be increased. The large delocalization energy could enhance the thermal stability of the polymer during HT.^25^

Covalent organic frameworks (COFs), as important two-dimensional porous polymers, play an indispensable role in the field of electrocatalysis.^26–28^ Metalloporphyrin-based COF (PorM-COF) has bifunctional oxygen catalysis performance. There are two different PorM-COF structures: (1) the general PorM-COF does not have a conjugated structure. Its electron delocalization is blocked, and its thermal stability is not improved.^29,30^ Thus, PorM-COF has poor catalytic performance when applied directly to an electrocatalytic reaction. PorM-COF usually needs to be pyrolyzed to form metallic-nitrogen-carbon clusters (M-N-Cs). The structure of M-N-Cs destroys the SAC structure of PorM-COF and has poor catalytic peformance.^31^ (2) A fully conjugated PorM-COF (FCPorM-COF) structure has a large π-bond with high delocalization energy, which leads to high thermal and chemical stability. FCPorM-COF has a lower HUMO-LUMO energy than monocyclic porphyrin, making it easy for it to gain and lose electrons in catalytic reactions.^32,33^ For example, FCPorM-COF, obtained by self-polymerization, has high thermal stability (does not decompose at 600 °C).^34^ Meanwhile, the fully conjugated imine-linked COF-366, azo-linked PorCo-COF,^35,36^ and C=C-linked sp^2^ C–COF all have excellent catalytic performance with high thermal stability. What kind of linkage should FCPorM-COF incorporate to achieve similarly outstanding oxygen catalysis performance?

FCPorM-COF with electron donor groups usually has excellent oxygen catalysis performance. The electron donor group can transfer electrons to the M–N_4_ center, which will increase the M–N_4_ electron cloud density and improve the oxygen catalysis performance of FCPorM-COF.^37,38^ Meanwhile, in previous reports, theoretical calculations have shown that a COF with a nitrogen-rich environment can accelerate the protonation process of intermediates in ORR,^39,40^ thereby improving the oxygen catalytic performance of the COF.^41^ Benzimidazole (BI) is a conjugated electron-rich heterocycle structure, which has a wide range of applications in the field of proton exchange membranes.^42,43^ BI is a typical electron donor group and can provide a nitrogen-rich environment for FCPorM-COF. The FCPorM-COF linked by BI groups should have the following advantages: (1) the molecular structure of FCPorM-COF forms a large π-bond with high delocalization energy, which improves the thermal stability of FCPorM-COF; (2) FCPorM-COF has a lower HUMO–LUMO energy than TCNPorCo, and its catalytic performance is enhanced; (3) electron-donating heterocycles enhance the catalytic performance of the M-N_4_ center; (4) the formation of a nitrogen-rich environment favors an adsorption mechanism for oxygen catalysis. To the best of our knowledge, there are no reports on BI group-linked cobalt porphyrin frameworks to enhance bifunctional oxygen electrocatalysis performance in the literature. Meanwhile, to improve the electronic conductivity of FCPorM-COF, it is often necessary to load FCPorM-COF onto conductive support materials.

FcPorM-COF has large π bonds, and internal π–π interactions make it form a tight topological structure.^17,44,45^ The topological structure of FcPorM-COF hinders its electron conductivity. Therefore, it is necessary to develop a conductive support for FCPorM-COF. Three-dimensional graphene (3D-G) is a porous graphite structure, which not only has the high conductivity of two-dimensional graphene but also has a high specific surface area and abundant defects. A recently reported carbon structure, 3D-G prepared by a hard template method, has a larger specific surface area, and its abundant pore structure provides an efficient mass transfer channel. In particular, the π bond on its surface can interact with the FcPorM-COF resulting in π–π interactions, synergistically promoting the oxygen catalysis reaction.^46^

Here, a fully conjugated two-dimensional poly(benzimidazole porphyrin-cobalt), abbreviated as PBIPorCo, has been synthesized *via* the polymerization reaction between meso-5,10,15,20-tetrakis(4-cyanophenylporphyrinato) cobalt (TCNPorCo) and 3,3′-diaminobenzidine (DAB). It was loaded onto three-dimensional graphene (3D-G) with a porous structure to increase the conductivity, specific surface area, and abundance of defects. UV-Vis, FT-IR, and nuclear magnetic resonance (NMR) studies proved the occurrence of the polymerization reaction, and the precise structure of the PBIPorCo. XRD, BET, and MS theoretical simulations were used to determine the pore structure and the topological structure configurations of the tetra-connected sql network. The micromorphology structure of the COF was characterized by HRTEM. HRTEM and powder X-ray diffraction (PXRD) were used to characterize the topological structure and exposed surface of the crystals. The chemical composition and valence states of the COF were characterized by X-ray photoelectron spectroscopy (XPS). TG was used to determine the thermal stability of the COF. To further investigate the oxygen catalysis process of the COF, density functional theory (DFT) was introduced to study the material using first-principles calculations.

## Results and discussion

### Synthesis and structural characterization of PBIPorCo

**Fig. 1:**
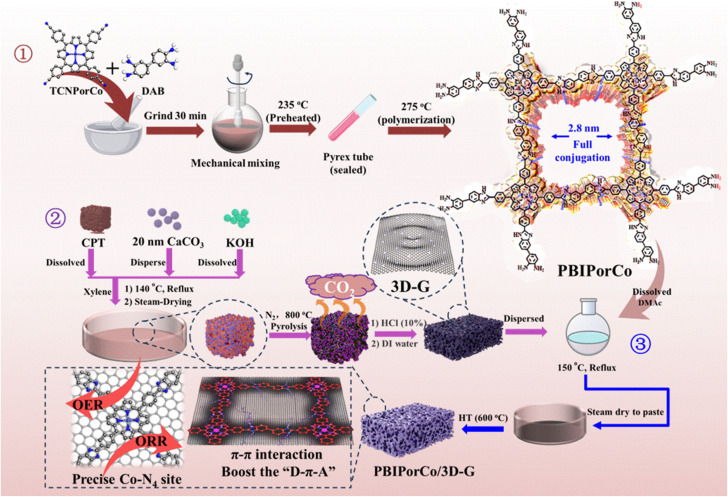
The preparation process of PBIPorCo and PBIPorCo/3D-G.

**Fig. 2:**
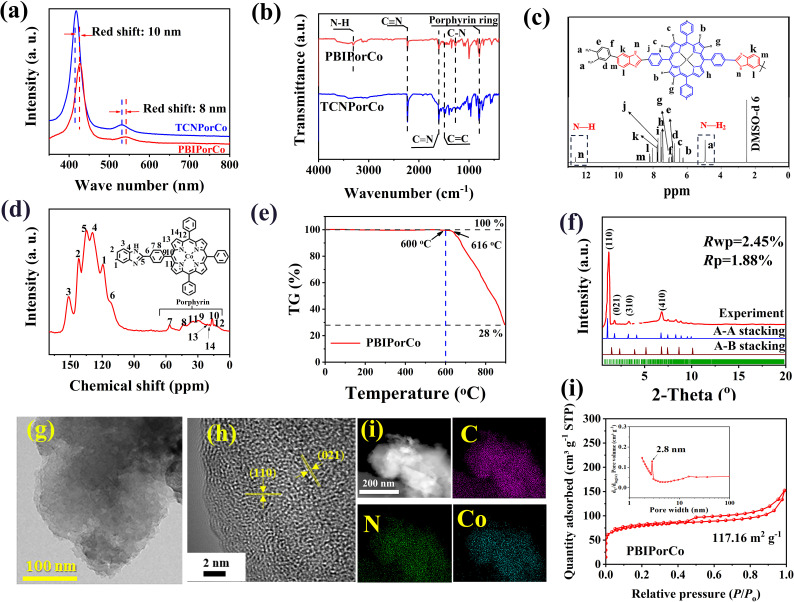
The characterization of PBIPorCo. (a) UV-Vis spectra of TCNPorCo and PBIPorCo; (b) FT-IR spectra; (c) ^1^H NMR spectrum of PBIPorCo (with the solvent DMSO-d6); (d) solid-state ^13^C cross-polarization magic-angle spinning (CP-MAS) nuclear magnetic resonance (NMR) spectrum; (e) TG analysis; (f) PXRD of PBIPorCo-600; (g) TEM; (h) HRTEM of PBIPorCo; (i) HAADF-STEM of PBIPorCo and mapping of C, N, and Co; (j) nitrogen adsorption–desorption isotherms and pore-size distribution plots for the samples.

PBIPorCo, the fully conjugated COF, was synthesized through a two-step polymerization process (Fig. 1, ①). The structural formula of PBIPorCo is shown in Fig. S1.† PBIPorCo was synthesized using metal porphyrin units with Co–N_4_ as the active center. Porphyrin cobalt(ii), commonly used as a standard sample for synchrotron radiation, is the structural unit of PBIPorCo. To compare the difference between the fully conjugated COF and the monocyclic metallic porphyrin, meso-5,10,15,20-tetra (benzimidazolyl phenyl) porphyrin cobalt (TBIPorCo) was synthesized in the same procedure as PBIPorCo (Fig. S2†). To characterize the structure of the prepared samples, ultraviolet-visible (UV-Vis) spectroscopy was used to identify the characteristic absorption peaks of the porphyrins. As shown in Fig. S3,† TCNPorH_2_ has five characteristic absorption peaks located at 418.8, 512.4, 551.2, 585.6, and 644.1 nm, of which the peak at 418.8 nm corresponds to the Soret characteristic absorption peak of porphyrin,^47^ and the other peaks are the characteristic absorption peaks of the Q band. The symmetry of the porphyrin is strengthened in the Co coordination reaction, and the number of Q-band absorption peaks reduced to one. The UV-Vis spectra (Fig. 2a) of TCNPorCo and PBIPorCo showed two characteristic absorption peaks, which are the Soret band (418.4 nm and 428.4 nm) caused by the π-to-π* transition in the conjugated system and the Q-band (530.2 nm and 540.2 nm), the characteristic peak of porphyrins.^48^ Due to the conjugation effect, the delocalization energy of the porphyrin plane increases, and the Soret band and Q-band of PBIPorCo redshift by 10 nm and 8 nm compared with TCNPorCo, respectively. TBIPorCo has Soret and Q band characteristic absorption peaks of a metal porphyrin (Fig. S4†). Compared with TCNPorCo, the Soret absorption peak and Q-band redshifted by 3 nm and 5 nm respectively, which proves that PBIPorCo continues to polymerize and its crystallinity increases after HT. The chemical structure of PBIPorCo was analyzed by Fourier transform infrared spectroscopy (FT-IR). As shown in Fig. 2b, there are six peaks located at 1350 cm^−1^ (C–N), 1600 cm^−1^ (C=C), 2220 cm^−1^ (–C≡N), 1650 cm^−1^ (–C=N), 1100 cm^−1^ (benzene rings), and 3440 cm^−1^ (–N–H). In addition, there was a relatively weak peak at 947 cm^−1^, which was caused by the bending vibration of the Co–N bonds. The vibrational strength of –C≡N in PBIPorCo was reduced, indicating that –C≡N was consumed during polymerization to form imidazole ring groups. The apparent presence of –N–H indicates the occurrence of polymerization.^49^ The –C≡N group is a strong electron withdrawing group, and the benzimidazole group is a strong electron donor group. The delocalization energy of the molecule increases after the introduction of the benzimidazole donor group. Fig. S5† shows that the –C≡N peak disappears, and the N–H peak appears, which proves the successful synthesis of TBIPorCo.

The chemical structure of PBIPorCo was further confirmed by ^1^H nuclear magnetic resonance spectra (^1^HNMR) and solid-state ^13^C cross-polarization magic-angle spinning (CP-MAS) NMR spectra. As shown in Fig. 2c, for ^1^H NMR of PBIPorCo (400 MHz) (DMSO-d, ppm), the peaks at *δ* 12.56 and 4.94 correspond to the N–H in BI and –NH_2_, respectively. The ^1^H NMR spectrum shows that the a-n positions correspond to H with peak positions of *δ* 4.94, *δ* 6.24, *δ* 6.44, *δ* 6.74, *δ* 6.84, *δ* 6.90, *δ* 7.07, *δ* 7.41, *δ* 7.53, *δ* 7.72, *δ* 7.76, *δ* 8.04, *δ* 8.19, and *δ* 12.56, respectively, which is consistent with the structural formula of PBIPorCo. The solid-state ^13^C NMR spectrum showed signals at *δ* 142, *δ* 151, *δ* 129, *δ* 134, *δ* 138, *δ* 112, *δ* 56, *δ* 43, *δ* 29, *δ* 16, *δ* 34, *δ* 14, *δ* 21, and *δ* 19 ppm, which could be assigned to carbon atoms 1–14, respectively, from the porphyrin and BI units of PBIPorCo (Fig. 2d). These results could provide further support for the successful formation of PBIPorCo. The N–H characteristic peak in the ^1^H NMR spectra (Fig. S6†) also verified the precise structure of TBIPorCo.

The thermogravimetry (TG) analysis (Fig. 2e) indicated that PBIPorCo is not decomposed at 600 °C. The crystallinity of the as-obtained COF was characterized by PXRD (Fig. 2f and S7†). Fig. S7† shows that PBIPorCo has low crystallinity, which will improve the solubility of PBIPorCo. To improve the crystallinity of PBIPorCo, PBIPorCo was heat treated for 2 h at 600 °C under a N_2_ atmosphere (PBIPorCo-600). Fig. S8† shows the UV-Vis spectrum of PBIPorCo-600 with a small redshift in the Soret and Q band characteristic absorption peaks. At the same time, the –C≡N peak decreases and N–H increases in the FT-IR spectrum (Fig. S9†), which proves that the polymerization continues at 600 °C. ^1^H NMR spectra (Fig. S10†) of PBIPorCo-600 shows that PBIPorCo does not decompose at 600 °C, and its structure does not change during HT. TG analysis shows that TBIPorCo decomposes at 395 °C and had lost 17% of its weight at 600 °C, which proved that the imidazole group was broken and BI groups were partially shed (Fig. S11†). PBIPorCo has a large conjugated structure and π bond, which provides PBIPorCo with a higher thermal stability than TBIPorCo.

As shown in the PXRD patterns for PBIPorCo-600 (Fig. 2f), the most intense peaks at 1.39°, 3.92°, 4.38°, 6.18°, 8.32°, 10.64°, 13.1°, 20.5°, and 21.3° were clearly observed, and were assigned to (001), (110), (020), (210), (300), (331), (231), (361), and (412) facets,^50^ respectively. Pawley refinements and spatial group information (see data files) were applied to investigate the theoretical structures with the self-consistent charge density functional tight binding (DFTB) method. PBIPorCo adopts an eclipsed stacking structure in a PM space group with refined cell parameters of *a* = 51.2 Å, *b* = 52.9 Å, and *c* = 5.21 Å. The experimental results were simulated with *R*_wp_ of 2.45% and *R*_p_ of 1.88%. Both eclipsed (A–A) and staggered (A–B) stacking (Fig. S12†) structures were simulated for the COF, and the simulated PXRD patterns of the eclipsed structures align well with the experimental results. Scanning electron microscopy (SEM) images of PBIPorCo demonstrate nanosheets with stacking blocks of solids, suggesting a stacked morphology of a class of two-dimensional polymers (Fig. S13†). Transmission electron microscopy (TEM) images further identified PBIPorCo as a densely stacked nanoscale sheet structure (Fig. 2g). In the high-resolution transmission electron microscopy (HR-TEM) image, the 0.3 nm d-spacing belongs to the (021) plane, and the 0.26 nm d-spacing was attributed to the (110) plane of PBIPorCo (Fig. 2h). In addition, high-angle annular dark-field scanning transmission electron microscopy (HAADF-STEM) and the corresponding Co, N, and C element mapping showed a uniform distribution of elements on the PBIPorCo (Fig. 2i). According to energy dispersive X-ray (EDX) plane scans, the atomic ratios of each element are close to those of the structure of PBIPorCo (Fig. S14†). The porous structures of the COF were studied using N_2_ physisorption measurements at 77 K. PBIPorCo displayed a IV-type structure (Fig. 2j), with a Brunauer–Emmett–Teller (BET) surface area of 117.16 m^2^ g^−1^ and pore sizes of 2.8 nm, which align well with the theoretical simulation of PBIPorCo (Fig. S15†). ICP-MS measurements revealed that the Co content in PBIPorCo was ∼3.74 wt%. (Table S1†).

### Synthesis and structural characterization of PBIPorCo/3D-G

**Fig. 3:**
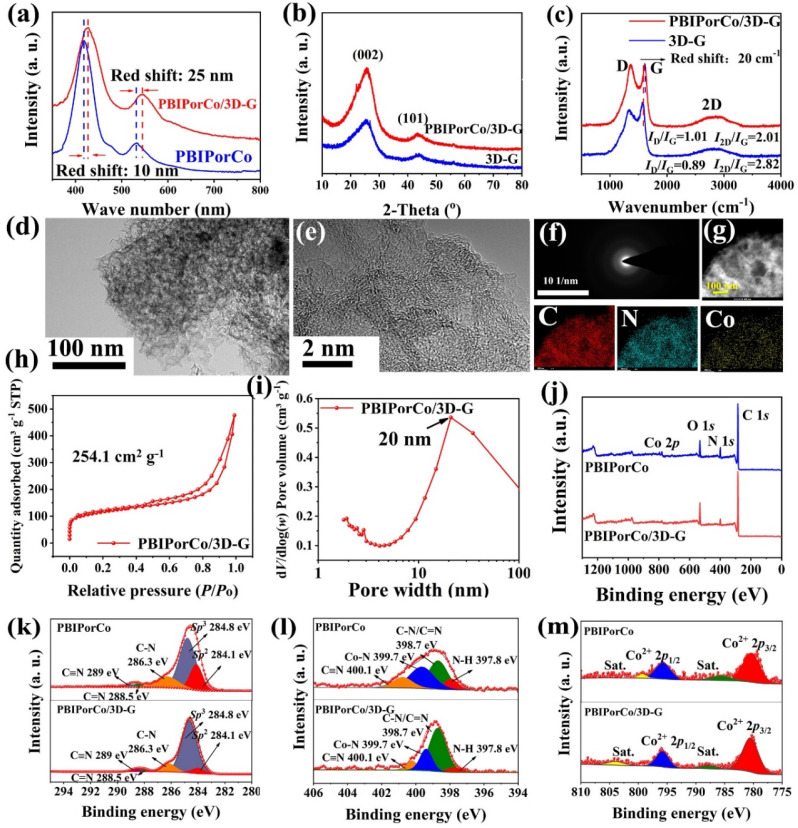
The characterization of oxygen catalysts. (a) UV-Vis diffuse reflectance absorption spectra; (b) XRD of PBIPorCo/3D-G and 3D-G; (c) Raman spectra; (d and e) TEM and HR-TEM of PBIPorCo/3D-G; (f) SAED; (g) HAADF-STEM and mapping of C, N, and Co; (h) BET surfaces; (i) pore size distribution; (j) XPS full spectra; (k–m) the high-resolution XPS spectra of PBIPorCo and PBIPorCo/3D-G: C 1s, N 1s, and Co 2p.

To further enhance the conductivity of the COF catalyst, PBIPorCo was anchored to the surface of three-dimensional graphene (3D-G) by the enhanced dipping method to obtain the PBIPorCo/3D-G (Fig. 1, ② and ③). HT was used to represent the activation process of the PBIPorCo/3D-G. The UV-Vis diffuse reflectance absorption spectra of the PBIPorCo/3D-G and the PBIPorCo are shown in Fig. 3a. The Soret band and Q band of PBIPorCo/3D-G, compared with PBIPorCo, were redshifted by 10 nm and 25 nm, respectively. This confirms the π–π interaction between PBIPorCo and 3D-G. In the powder XRD (PXRD) spectra (Fig. 3b), PBIPorCo/3D-G showed only the characteristic diffuse reflection peaks of graphene and no other obvious peaks when compared with 3D-G. There were two characteristic peaks at 25° and 43°, which were attributed to the (110) and (103) planes of graphene. This suggests that: (1) PBIPorCo lies flat on the surface of 3D-G and no stacking occurs; (2) PBIPorCo is not decomposed during the HT of the PBIPorCo/3D-G catalyst. The Raman spectra of PBIPorCo/3D-G and 3D-G (Fig. 3c) revealed that the D and G peaks represent the extent of defects and graphitization in sp^2^ carbon, respectively. The *I*_D_/*I*_G_ values for PBIPorCo/3D-G and 3D-G are 1.01 and 0.89, respectively. PBIPorCo/3D-G had a more abundant defect structure than 3D-G, which was more conducive to exposing the active sites. Meanwhile, no absorption peaks of PBIPorCo stacking were detected on the catalyst surface. Notably, the G-peak in PBIPorCo/3D-G experiences a significant redshift (20 cm^−1^) compared to 3D-G due to π–π interaction between PBIPorCo and 3D-G (Fig. S16†).^51^

The SEM images of PBIPorCo/3D-G (Fig. S17†) shows a foam-like carbon structure with multi-level pore channels. The PBIPorCo/3D-G exhibited a foamy porous structure, as seen by the TEM images (Fig. 3d). Additionally, the HR-TEM (Fig. 3e) shows a typical graphene-like structure without any visible lattice stripes, suggesting that PBIPorCo distributes uniformly on the 3D-G surface without decomposition to form oxides, nitrides or atoms clusters of Co–N–C. The SAED of PBIPorCo/3D-G demonstrated that there are no diffraction spots of Co atom clusters, and it is an amorphous structure (Fig. 3f). EDS showed that the catalyst contained C, N, and Co elements, and its contents matched the theoretical ratio of PBIPorCo to PBIPorCo/3D-G (Fig. S18†). HAADF-STEM further illustrated the uniform dispersion of C, N, and Co (Fig. 3g). The results show that PBIPorCo is uniformly distributed on the 3D-G surface.

The specific surface area and pore size distribution are shown in Fig. 3h and i, and PBIPorCo/3D-G exhibites a surface area of 254.1 m^2^ g^−1^. The mesoporous and microporous structures are abundant in PBIPorCo/3D-G due to CO_2_ decomposition by CaCO_3_ during the pyrolysis process, resulting in multi-level pore structure formation. Additionally, there is a distinct characteristic peak at around 20 nm attributed to mesopores generated by template CaCO_3_ (Fig. 3i). As is shown in Table S1,† the total Co content in the PBIPorCo/3D-G electrocatalyst is about ∼1.63 wt%. The surface chemistry phase and composition of PBIPorCo and PBIPorCo/3D-G were analyzed by XPS, revealing the presence of C, N, and Co elements (Fig. 3j and Table S2†), showing that the composition and proportion of each element are similar between PBIPorCo and PBIPorCo/3D-G, which proves that PBIPorCo does not decompose during HT. The ratio of Co in PBIPorCo and PBIPorCo/3D-G is close to 2 : 1, which is consistent with the ratio of PBIPorCo and 3D-G. The back-convolution of the C 1s signals produces peaks at 284.1, 284.8, 286.3, 288.5, and 289 eV, corresponding to C sp^2^, C sp^3^, C–N, C=N and C≡N, respectively (Fig. 3k). Similarly, the high-resolution N 1s spectrum consists of four peaks at 397.8, 398.7, 399.7, and 400.1 eV for N–H, C–N/C=N, Co–N, and C≡N, respectively (Fig. 3l). In addition, two sets of peaks at 780.4/795.5 eV in the Co 2p XPS spectra indicate that the cobalt in PBIPorCo and PBIPorCo/3D-G was in an intermediate oxidation state with Co^2+^ (Fig. 3m). The XPS spectrum of PBIPorCo/3D-G compared with that of PBIPorCo showed that the chemical states of each element were stable after HT, which proved that PBIPorCo remained intact during the whole HT process. Combined with TG, XPS, HRTEM, Raman, and PXRD analysis, it can be concluded that PBIPorCo does not decompose when it adheres to 3D-G through π–π interaction. Therefore, the catalytically active center of the catalyst is Co–N_4_. PBIPorCo has been proven to be a pure compound rather than a composite, with a fixed structure. PBIPorCo retains its original structure and lies on the 3D-G surface with a well-defined Co–N_4_ coordination environment.

### Bifunctional oxygen catalytic performance evaluation

**Fig. 4:**
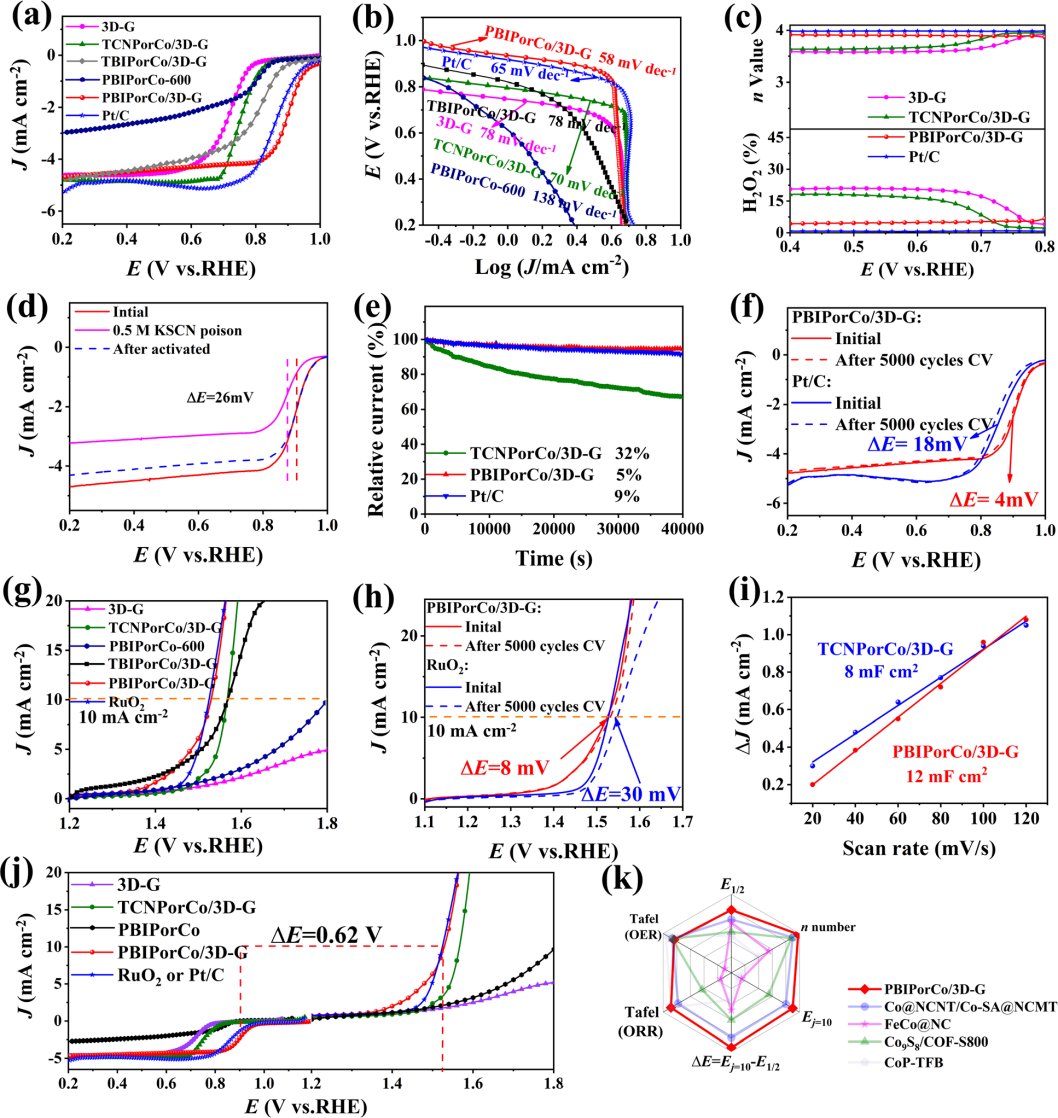
The ORR test and OER performance evaluation. (a) LSV curves; (b) Tafel curves; (c) the number of electrons transferred and H_2_O_2_ yields obtained using a RRDE; (d) the poisoning experiments for PBIPorCo/3D-G (O_2_-saturated 0.1 M KOH) with and without 0.05 M KSCN; (e) *i*–*t* curve; (f) accelerated durability testing (ADT) curves; (g) LSV curves; (h) ADT curves; (i) *C*_dl_ calculation; (j) Δ*E* calculation for ORR and OER; (k) comparison of half-wave potential (*E*_1/2_), n number, *E*_*j* = 10_, Δ*E*, Tafel value (ORR), and Tafel value (OER) for PBIPorCo/3D-G and the other COF materials.

The ORR catalyst performance studies were focused on the investigation of the HT temperature and preparation ratio of PBIPorCo/3D-G, aiming to determine the optimal process conditions (Fig. S19†). In a series of samples prepared, an optimal activation temperature of 600 °C and an optimal ratio of 1 : 1 were determined, and PBIPorCo/3D-G was the best performing catalyst. Cyclic voltammetry (CV) tests (Fig. S20†) were conducted on PBIPorCo/3D-G, TCNPorCo/3D-G, 3D-G, and Pt/C under N_2_/O_2_ saturation conditions in 0.1 mol L^−1^ KOH. The PBIPorCo/3D-G exhibited a higher reduction potential than Pt/C under O_2_-saturation. In the linear sweep voltammetry (LSV) curves (Fig. 4a), the half-wave potentials of PBIPorCo/3D-G, PBIPorCo-600, TBIPorCo/3D-G, TCNPorCo/3D-G, 3D-G, and Pt/C were measured as 0.90, 0.79, 0.81, 0.78, 0.70, and 0.86 V *vs.* RHE, respectively. Furthermore, the Tafel curve of PBIPorCo/3D-G demonstrated a lower slope (58 mV dec^−1^), indicating it had a faster dynamic ORR process than Pt/C (65 mV dec^−1^) (Fig. 4b). The results show that PBIPorCo/3D-G had the highest half-wave potential and a quick oxygen catalysis kinetic process compared to the other materials. A full-speed ORR test was conducted on the PBIPorCo/3D-G (Fig. S21†), TCNPorCo/3D-G, 3D-G, and Pt/C (Fig. S22†), and the K–L curves were fitted to determine the electron transfer number. The electron transfer numbers for PBIPorCo/3D-G, TCNPorCo/3D-G, and 3D-G are 3.9, 3.7, and 3.4, respectively (Fig. S23†). Similarly, accurate electron transfer numbers calculated using a rotating ring disk electrode (RRDE) were determined as 3.98, 3.85, 3.75, and 4.00 for PBIPorCo/3D-G, TCNPorCo/3D-G, 3D-G, and Pt/C, respectively (Fig. 4c). A ring galvanometer was used to calculate the H_2_O_2_ generation rate at different potentials, which represents electrochemical *in situ* analysis of the kinetic process at different potentials.

Fig. 4d shows that after poisoning experiments, *E*_1/2_ of PBIPorCo/3D-G decreased from 0.90 to 0.85 V *vs.* RHE. Furthermore, it was observed that under O_2_-saturation conditions, the oxygen reduction activity of PBIPorCo could be fully recovered. [SCN]^−^ can form complexes with Co^2+^, providing evidence for the presence of the Co–N_4_ site through poisoning experiments. The results of the poisoning experiments proved that PBIPorCo/3D-G is a SAC. The real active site was determined as Co–N_4_ in the PBIPorCo molecule. The chronoamperometry curves (Fig. 4e) compared the durability of PBIPorCo/3D-G, TCNPorCo/3D-G, and Pt/C at a constant potential (0.5 V *vs.* RHE), and current densities decreased by 5%, 32%, and 9%, respectively, were observed. Fig. 4f shows accelerated durability testing (ADT), where *E*_1/2_ values of PBIPorCo/3D-G and Pt/C are attenuated by only 9 mV and 15 mV, respectively, after 5000 CV cycles. However, the *E*_1/2_ of TCNPorCo/3D-G has a negative shift of 16 mV and is not as durable as PBIPorCo/3D-G (Fig. S24†). The *i*–*t* and ADT results show the durability of PBIPorCo/3D-G for ORR, which is attributed to the fully conjugated structure of PBIPorCo. This conjugated structure increases the delocalization energy within PorM, thereby improving its chemical stability, which further enhances the electrochemical stability of PBIPorCo/3D-G. PBIPorCo/3D-G and Pt/C were tested in oxygen-saturated 0.1 M KOH. Methanol solution was added at 200 min. As shown in Fig. S25,† the *i*–*t* curve of PBIPorCo/3D-G was unaffected by methanol addition, while the current of Pt/C was attenuated significantly. This indicates that PBIPorCo/3D-G has good methanol tolerance. ORR tests were performed on PBIPorCo/3D-G and Pt/C in oxygen-saturated 0.5 M H_2_SO_4_, and the *E*_1/2_ of PBIPorCo/3D-G and Pt/C were 0.82 and 0.79 V *vs.* RHE, respectively. Therefore, PBIPorCo/3D-G has good ORR performance under acidic conditions (Fig. S26†). EIS tests were performed for ORR of PBIPorCo/3D-G and Pt/C. Fig. S27† shows the EIS curves at 0.85 V *vs.* RHE (the illustration shows the equivalent circuit). PBIPorCo/3D-G has a smaller internal resistance and reaction internal resistance.

The OER performances of the catalysts were tested in 1 M KOH solution (Fig. 4g), and the potentials at a current density of 10 mA cm^−2^ of the PBIPorCo/3D-G, PBIPorCo-600, TBIPorCo/3D-G, TCNPorCo/3D-G, 3D-G, and RuO_2_ catalysts are 1.52, 1.80, 1.56, 1.56, 1.95, and 1.50 V *vs.* RHE, respectively. The overpotentials of the samples are 290, 570, 330, 330, 720, and 270 mV, respectively. Notably, PBIPorCo/3D-G demonstrated the smallest Tafel slope (108 mV dec^−1^) compared to RuO_2_ (138 mV dec^−1^), PBIPorCo-600 (180 mV dec^−1^), TBIPorCo/3D-G (128 mV dec^−1^) and TCNPorCo/3D-G (167 mV dec^−1^), indicating it had superior kinetic activity (Fig. S28†). The oxygen catalytic performance enhancement of PBIPorCo/3D-G is due to the strong π–π interaction between PBIPorCo and 3D-G compared to 3D-G alone. Due to the poor thermal stability and the loss of edge electron donor groups, the oxygen-catalyzed reaction of TBIPorCo/3D-G has low performance. However, its oxygen catalysis reaction performance is better than that of TCNPorCo/3D-G, which is due to the low content of BI electron donor groups. In the chronoamperometry curve (Fig. S29†), PBIPorCo/3D-G experienced only a current decrease of 14%, while TCNPorCo/3D-G, 3D-G, and Pt/C demonstrated a decrease of approximately 27%, 36%, and 23%, respectively. Moreover, for the ADT, after 5000 CV cycles (Fig. 4h), PBIPorCo/3D-G exhibited an overpotential shift of 8 mV, which was lower than those for RuO_2_ (30 mV) and the TCNPorCo/3D-G (20 mV) (Fig. S30†). Fig. 4i shows the double-layer capacitance (*C*_dl_) (Fig. S31†), and the slope for PBIPorCo/3D-G was 12 mF cm^−2^, which was higher than that for TCNPorCo/3D-G, confirming its larger electrochemical specific surface area (ECSA). The bifunctionality analysis (Fig. 4j), represented by Δ*E* = *E*_*j* = 10_ – *E*_1/2_, reveals that PBIPorCo/3D-G possesses a Δ*E* value of 0.62 V, which is superior to those of Pt/C (Δ*E* = 0.90 V) and RuO_2_ (Δ*E* = 0.88 V). Therefore, PBIPorCo/3D-G demonstrates rapid oxygen catalysis kinetics, the highest half-wave potential, the lowest overpotential (*E*_*j* = 10_), and the most efficient bifunctional oxygen catalysis performance (Δ*E*) (Fig. 4k). Due to the poor thermal stability and the loss of edge electron donor groups, TBIPorCo/3D-G had a low oxygen catalysis reaction performance. TBIPorCo/3D-G exhibits a better performance than TCNPorCo/3D-G, which is due to the low content of electron donor groups. Therefore, the excellent oxygen catalysis performance of PBIPorCo/3D-G can be attributed to the formation of fully conjugated large π bonds in PBIPorCo and the presence of benzimidazole groups, which act as strong electron donor groups.

### Composition, morphology, and structure characterization after durability testing

**Fig. 5:**
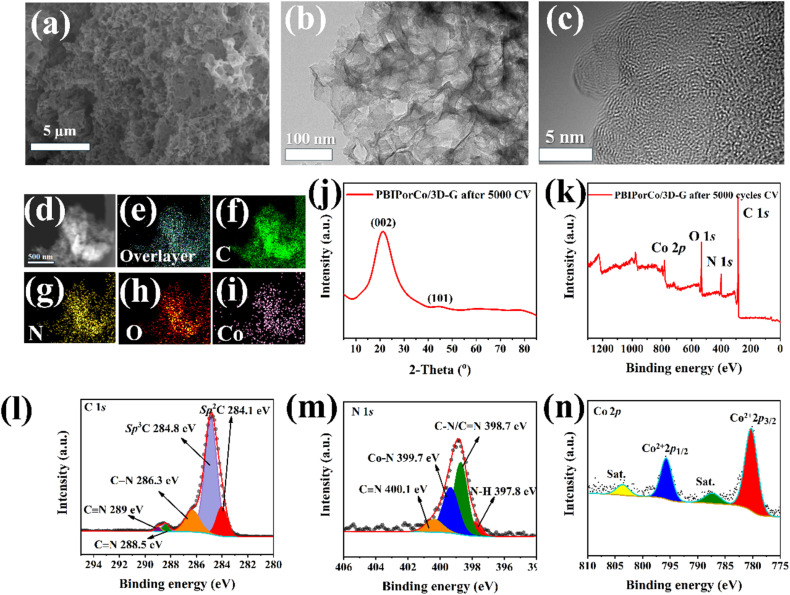
Composition, morphology, structure and valence state characterization after durability testing. (a) SEM of PBIPorCo/3D-G-5000; (b and c) TEM and HR-TEM of PBIPorCo/3D-G-5000; (d) HAADF-STEM; (e–i) mapping of overlayer, C, N, O, and Co; (j) XRD of PBIPorCo/3D-G-5000; (k) XPS full spectra; (l–n) high-resolution XPS spectra of PBIPorCo/3D-G-5000: C 1s, N 1s, and Co 2p.

The PBIPorCo/3D-G after 5000 CV cycles (PBIPorCo/3D-G-5000) was analyzed to verify the chemical stability of the PBIPorCo/3D-G. Fig. 5a depicts the SEM of PBIPorCo/3D-G-5000, revealing that its porous configuration remains intact and is not compromised during the electrocatalytic reaction. Fig. 5b and c present the TEM and HR-TEM, respectively, of PBIPorCo/3D-G-5000. After undergoing electrocatalytic testing, PBIPorCo retained a foam-like graphene structure. In the HRTEM images, no lattice of atomic clusters or metal oxidation were observed in PBIPorCo/3D-G, indicating that it retains an amorphous carbon structure. The staggered distribution of elements in HAADF-STEM (Fig. 5d) and the uniform distribution of elements in the mapping (Fig. 5e–i) could be observed. Fig. 5j shows that the XRD of PBIPorCo/3D-G-5000 still represents amorphous diffuse reflection. Fig. 5k presents the full XPS spectrum of PBIPorCo/3D-G-5000, indicating that all elements remained stably present. To further analyze the valence states of each element, the deconvoluted peaks of C 1s, N 1s, and Co 2p were examined (Table S3†). Each element retains its structure after the oxygen catalysis reaction, and both the existence state and valence state of each element remain unchanged. The above results are consistent with the structure both before and after the CV test, thereby demonstrating that the structure of PBIPorCo remained stable in the durability testing and exhibited high chemical stability. The ORR reaction only involves the center of Co–N_4_ in the PBIPorCo molecule, and the other group of the PBIPorCo molecule has not changed.

### DFT calculations

**Fig. 6:**
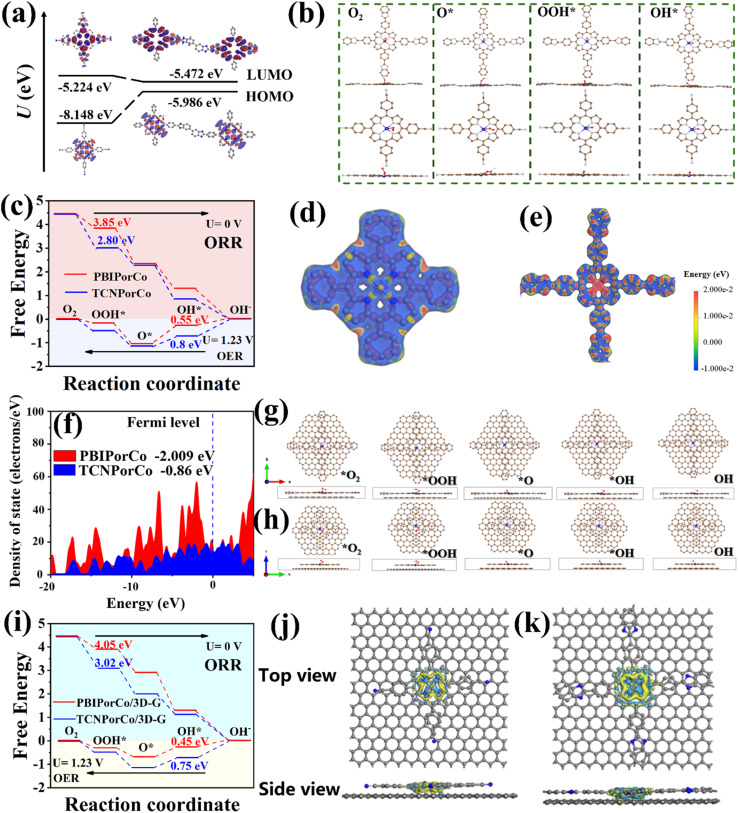
DFT calculations. (a) The energy levels of the HOMO and LUMO of TCNPorCo and PBIPorCo; (b) PBIPorCo and TCNPorCo adsorption intermediate models (Co–N_4_ site); (c) Gibbs free energy change (Δ*G*) of TCNPorCo and PBIPorCo after ORR and OER; (d and e) electrostatic potential (ESP) of TCNPorCo and PBIPorCo; (f) DOS of TCNPorCo and PBIPorCo; (g and h) adsorption oxygen intermediate models for the PBIPorCo/3D-G and PorCo/3D-G repeating unit (Co–N_4_ site); (i) Gibbs free energy change (Δ*G*) of TCNPorCo and PBIPorCo after ORR and OER; (j and k) charge density difference of PBIPorCo/3D-G and PorCo/3D-G.

To further understand the catalytic mechanism of PBIPorCo, HOMO–LUMO orbits of PBIPorCo and TCNPorCo were simulated by Materials Studios (Ms 2019, DMOl 3 module). As shown in Fig. 6a, the energy gap between the HOMO and LUMO of TCNPorCo was 2.924 eV, and that of PBIPorCo was 0.514 eV. It can be seen that the electron leap of PBIPorCo was less than that required for TCNPorCo, suggesting that PBIPorCo will have a smaller energy gap and be more likely to lose or gain electrons to effectively catalyze ORR and OER. DFT calculations show an in-depth understanding of the effects of BI and Co–N_4_ sites in PBIPorCo during the catalytic process (Fig. 6b). The Gibbs free energy (*G*) is shown in Fig. 6c, and the rate-limiting step of the ORR on both Co–N_4_ sites was the generation of the *OOH intermediate by the protonation of the adsorbed *O_2_ and the *OH intermediate. Compared with the PorCo, the PBIPorCo exhibited a lower energy barrier for the rate-limiting step, thus resulting in its higher activity. This indicates that the nitrogen-rich environment of PBIPorCo was more conducive to the protonation process, which is sufficient to enhance the catalytic ORR process. Electrostatic potential (ESP) analysis demonstrates that the N electron cloud on the BI was shifted towards the centrally coordinated Co–N_4_ (Fig. 6d and e). The electron cloud density of Co–N_4_ increased from ∼0.005 eV to ∼0.02 eV. This indicated that the linkage of the BI serves as a donor of electrons, enhances the electron cloud density of Co–N_4_, and improves the catalyzed ORR performance of the PBIPorCo (Fig. 6g). The density of states (DOS) and band structure of TCNPorCo and PBIPorCo were calculated using the CASTEP module in Material Studios (Fig. 6f), and PBIPorCo had a lower d-band center compared to TCNPorCo. This indicated the weakening of the binding effect between the Co–N_4_ and the intermediate, which was beneficial to the desorption process and accelerated the oxygen catalysis process.

Both PBIPorCo and TCNPorCo had forbidden bands in their band energy structures (Fig. S32†). However, the forbidden band of PBIPorCo (0.0436 eV) was smaller than that of TCNPorCo (0.1415 eV), indicating that PBIPorCo had a fully conjugated structure with superior electron transfer properties compared to those of TCNPorCo. Oxygen catalysis adsorption intermediate models of PorCo/3D-G (Fig. 6g) and PBIPorCo/3D-G (Fig. 6h) were established to study the internal catalytic mechanism of the catalysts. The adsorption free energy trend of PBIPorCo/3D-G for oxygen intermediates was similar to that of PBIPorCo and a smaller free energy was exhibited. Compared with PBIPorCo, PBIPorCo/3D-G exhibits rapid protonation and stronger adsorption to *O (Fig. 6i), so it has a better oxygen catalysis effect, which is consistent with the experimental results. These results prove the π–π interaction of 3D-G with PBIPorCo and the electron transport mechanism inside PBIPorCo/3D-G. The charge density difference is calculated by the CASTEP module, and the yellow and blue electron distributions represent the processes of electron gain and loss, respectively (Fig. 6j and k). The electron-rich 3D-G can interact with PBIPorCo and transfer electrons to the Co–N_4_ site to form an “electron donor-electron acceptor” structure, which collaboratively improves the oxygen catalysis process of PBIPorCo/3D-G. The reasons for the enhanced performance of PBIPorCo are as follows: (1) PBIPorCo has a lower HOMO–LUMO energy than TCNPorCo, which is conducive to the easy gain and loss of electrons in ORR and OER; (2) the nitrogen-rich environment of PBIPorCo is more favorable for the protonation process in oxygen catalysis, and is sufficient to enhance the catalyzed ORR process; (3) the metalloporphyrin unit in PBIPorCo relied on the BI group linkage, and the electron-donating BI can transfer electrons to the Co–N_4_ sites, further enhancing the catalytic activity of the COF catalyst; (4) the d-band center of PBIPorCo is lower than the Fermi energy level, which promotes the desorption of oxygen intermediates; (5) the π–π interaction between 3D-G and PBIPorCo could transfer electrons to the Co–N_4_ site as an “electron donor-electron acceptor” structure, which collaboratively enhances the oxygen catalysis process; (6) a small forbidden band gap exists in the energy band structure of PBIPorCo, which proves its better electronic conductivity.

### Zn–air battery performance evaluation

**Fig. 7:**
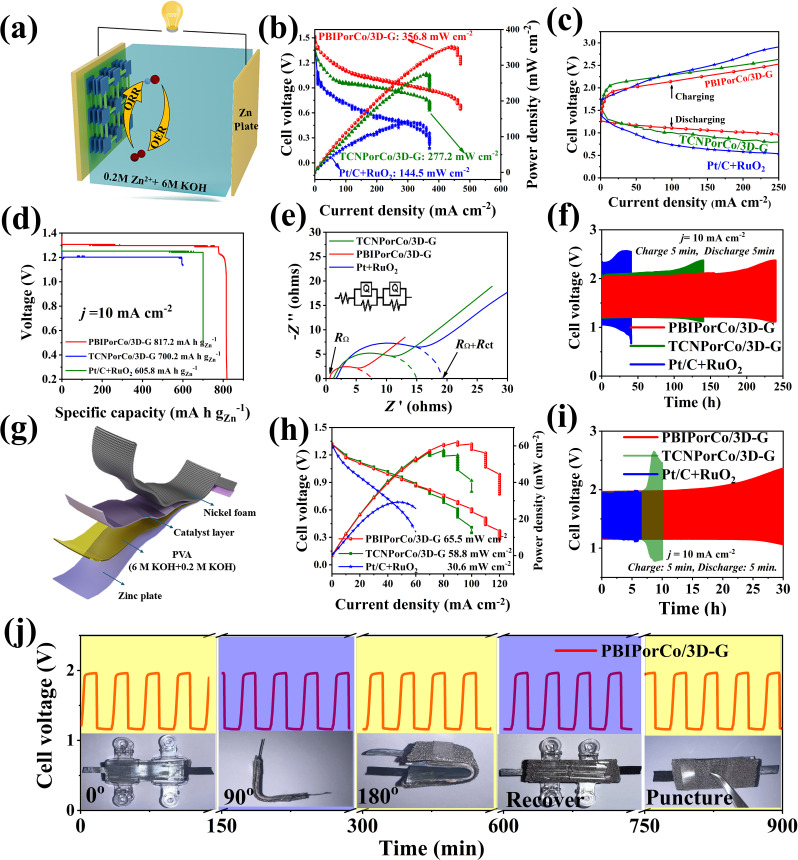
Zn–air battery performance evaluation. (a) Diagram of a model of L-ZABs; (b) discharge curves of L-ZABs; (c) charge and discharge polarization curves of a Zn–air battery; (d) specific capacity of L-ZABs (∼10 mA cm^−2^); (e) EIS testing of L-ZABs; (f) cyclic stability performance curves of L-ZABs at 10 mA cm^−2^; (g) schematic diagram of F-ZABs; (h) power density; (i) cycling stability of the solid flexible Zn–air battery; (j) galvanostatic discharge and charge curves of the Zn–air battery with the PBIPorCo/3D-G cathode under bending.

ZABs are important devices to test the bifunctional performance of PBIPorCo/3D-G, and its charging and discharging performance corresponds to ORR and OER, respectively. Liquid zinc–air batteries (L-ZABs) were assembled with a cathode gas diffusion layer coated with PBIPorCo/3D-G (Fig. 7a). In Fig. 7b, L-ZABs equipped with PBIPorCo/3D-G demonstrated a peak power density of 356.8 mW cm^−2^, which was higher than those of TCNPorCo/3D-G (277.2 mW cm^−2^) and Pt/C + RuO_2_ (144.5 mW cm^−2^). The charge–discharge polarization curves are shown in Fig. 7c. The charge and discharge gap of PBIPorCo/3D-G (1.00 V) was smaller than those of TCNPorCo/3D-G (1.25 V) and Pt/C + RuO_2_ (1.50 V) at 100 mA cm^−2^. The L-ZABs equipped with PBIPorCo/3D-G were step-discharged at different currents (10, 20, 30, 40, and 50 mA cm^−2^), and the voltage was stable during the current variation, which proved the stable structure of the PBIPorCo/3D-G in the L-ZABs discharge process (Fig. S33†). The L-ZABs equipped with PBIPorCo/3D-G, TCNPorCo/3D-G, and Pt + RuO_2_ were discharged at a constant current of 10 mA cm^−2^ (Fig. 7d), demonstrating specific capacity performances of 817.2, 700.2, and 605.8 mA h g_Zn_^−1^. Fig. 7e shows the electrochemical impedance spectra of several L-ZABs, with *R*_Ω_ representing the impedance of the materials and electrolytes in the L-ZABs and *R*_ct_ representing the interfacial transfer resistance. The arc radius is the sum of the internal resistance and the interfacial transfer resistance. The resistance (*R*_Ω_) of the L-ZAB containing PBIPorCo/3D-G was 1 Ω and the interfacial reaction resistance (*R*_ct_) was 6.5 Ω. Both *R*_Ω_ and *R*_ct_ of L-ZAB containing PBIPorCo/3D-G were smaller than those of a ZAB containing TCNPorCo/3D-G (*R*_Ω_ = 2.0 Ω, *R*_ct_ = 13.0 Ω) and L-ZAB assembled with Pt/C + RuO_2_ (*R*_Ω_ = 2.3 Ω, *R*_ct_ = 17.0 Ω). The diffusion process in the Nyquist plot of PBIPorCo/3D-G was closest to 45°, which proved that it has the fastest charge transfer process and mass transfer process. Two L-ZABs successfully light the “SDUT” shaped LEDs (Fig. S34†). Fig. 7f demonstrates the charge–discharge cycle stability of L-ZABs, where PBIPorCo/3D-G was used for over 200 h at a charging and discharging current density of 10 mA cm^−2^, exhibiting superior stability compared to PtC + RuO_2_ (25 h). The performance of PBIPorCo/3D-G in L-ZABs is better than that in previous reports (Table S4†).

Flexible all-solid zinc–air batteries (F-ZABs) were assembled with PBIPorCo/3D-G as the cathode gas diffusion layer (Fig. 7g). F-ZABs equipped with PBIPorCo/3D-G can reach a peak power of 65.5 mW cm^−2^, compared with TCNPorCo/3D-G and Pt/C + RuO_2_, which can only reach 58.8 and 30.6 mW cm^−2^ (Fig. 7h). The charge–discharge polarization curve is shown in Fig. S35.† The charge and discharge gap of PBIPorCo/3D-G was 0.80 V, which was smaller than those of Pt/C + RuO_2_ (1.00 V) and TCNPorCo/3D-G (1.20 V) at 50 mA cm^−2^. Two F-ZABs can light up an “SDUT” shaped LED (Fig. S34†). The PBIPorCo/3D-G and Pt/C F-ZABs were tested with a specific capacity of 10 mA cm^−2^, and the PBIPorCo/3D-G based battery could remain stable for 36 h, while the Pt/C + RuO_2_-based F-ZABs could only reach 29 h. Therefore, PBIPorCo/3D-G-based F-ZABs have good F-ZAB performance (Fig. S36†). Fig. 7i shows the charge–discharge cycle stability of F-ZABs. PBIPorCo/3D-G can be tested for more than 25 h at 10 mA cm^−2^ charging current and discharge current, which represented greater stability than TCNPorCo/3D-G (8 h) and PtC + RuO_2_ (5 h). Notably, the voltage gap (Fig. 7j) presented negligible change when the flexible battery was bent from an angle of 0° to 180°, indicating the practicability in the fields of portable and wearable electronics. These results demonstrate the high potential of PBIPorCo/3D-G based all-solid-state ZABs for future flexible electronic devices.

## Experimental

### Synthesis of poly(benzimidazole porphyrin-cobalt) (PBIPorCo)

A mixture of TCNPorCo (0.5 mmol, 0.386 g) and 3,3-diaminobenzidine (DAB) (1.2 mmol, 0.258 g) was ground in the solid phase and mechanically stirred for 10 min to obtain a homogeneous powder. Subsequently, the powder was melted and preheated at 235 °C for 3 h under the protection of N_2_. The polymerization of the intermediate was continued at 275 °C for 3 h. The coarse products obtained were immersed in a mixture of ethanol and DI water (*V*_ethanol_ : *V*_DI water_ = 7 : 3), filtered to obtain a filter cake, and purified by recrystallization with DMF three times, and the purplish-black products obtained were dried and named PBIPorCo (0.486 g, yield ∼ 62.9%).

To improve the crystallinity of PBIPorCo, it was heat treated for 2 h at 600 °C under a nitrogen atmosphere and named PBIPorCo-600.

### Synthesis of meso-5,10,15,20-tetra (benzimidazolyl phenyl) porphyrin Co(ii) (TBIPorCo)

A mixture of TCNPorCo (0.5 mmol, 0.386 g) and *O*-phenylenediamine (DAB) (2.2 mmol, 0.238 g) was ground in the solid phase and mechanically stirred for 10 min to obtain a homogeneous powder. Subsequently, the powder was melted and preheated at 235 °C for 3 h under the protection of N_2_. The polymerization of the intermediate was continued at 275 °C for 3 h in a sealed pyrex tube. The coarse products obtained were immersed in a mixture of ethanol and DI water (*V*_ethanol_ : *V*_DI_ water = 7 : 3), filtered to obtain a filter cake, and purified by recrystallization with DMF three times, and the purplish-black products obtained were dried and named meso-5,10,15,20-tetra(benzimidazolyl phenyl) porphyrin Co(ii) (TBIPorCo) (0.389 g, yield ∼ 68.6%).

### Preparation of PBIPorCo/3D-G

0.1 g of PBIPorCo was dissolved in 90 mL of *N*′*N*-dimethylacetamide (DMAc) heated to 175 °C. 0.1 g of 3D-G was dispersed in 90 mL of DMAc, added into the above mixture solution, and refluxed for 24 h. DMAc was removed by distillation under reduced pressure to give a black paste precursor. The black precursor was dried under vacuum at 60 °C for 12 h to obtain a black solid. The solid powder was ground and heat-treated at 600 °C protected under a N_2_ atmosphere for 2 h. The product was named PBIPorCo/3D-G.

TCNPorCo/3D-G and TBIPorCo/3D-G were also prepared in a similar way.

## Conclusions

PBIPorCo has been successfully synthesized as a fully conjugated COF, exhibiting high thermal stability as confirmed by UV-Vis, FT-IR, and NMR analyses. Topological simulations and PXRD results indicate that PBIPorCo predominantly exhibits an A–A structure. DFT calculations reveal that PBIPorCo has a lower HUMO–LUMO energy than TCNPorCo and can easily gain and lose electrons. The incorporation of electron-donating BI group can increase the electron cloud density of Co–N_4_ and enhance the oxygen catalytic activity of PBIPorCo. In addition, the nitrogen-rich structure promotes protonation during ORR. PBIPorCo forms a π–π interaction with 3D-G, adapting an “electron donor–electron acceptor” configuration, which can synergistically enhance the oxygen catalytic activity. HR-TEM, XRD, and Raman studies demonstrate that PBIPorCo is anchored on the 3D-G surface. PBIPorCo/3D-G has excellent oxygen catalytic activity, with *E*_1/2_ = 0.90 V *vs.* RHE, and *E*_*j* = 10_ = 1.52 V *vs.* RHE/*η*_*j* = 10_ = 290 mV. The highly efficient bifunctional oxygen catalyst (Δ*E* = 0.62 V) is expected to be used in FCs and OWS. L-ZABs and F-ZABs equipped with PBIPorCo/3D-G can achieve power densities of 356.8 and 65.5 mW cm^−2^, respectively. This work provides novel design strategies for fully conjugated COFs in electrocatalytic applications.

## Data availability

The details of the experimental procedures, X-ray diffraction, and computational methods and details are provided in the ESI.†

## Author contributions

Y. S: investigation, writing – original draft, formal analysis, software. W. D: data curation, investigation, methodology. J. W and P. S.: resources, methodology, writing – review & editing. Y. Z: resources, methodology. Z. L: conceptualization, supervision, validation, writing-review & editing, funding acquisition.

## Conflicts of interest

There are no conflicts to declare.

## Supplementary Material

SC-016-D5SC02082D-s001
