# Supplementary material for: A novel fully conjugated COF adorned on 3D-G to boost the “D–π–A” electron regulation in oxygen catalysis performance
Source: Chem Sci. 2025 Apr 22;16(22):9951–65. doi: 10.1039/d5sc02082d (PMC12053216; doi:10.1039/d5sc02082d)
Supplement: SC-016-D5SC02082D-s001 [file SC-016-D5SC02082D-s001.pdf]

Supplementary Information (SI) for Chemical Science.
This journal is © The Royal Society of Chemistry 2025

Supporting Information

Novel Fully Conjugated COF Adorned on 3D-G to Boost the "D-π-A" Electron Regulation in Oxygen Catalysis Performance

*Ying-gang Sun[a], Wen-jie Duan[a], Ji-gang Wang[a], Peng Sun[a], Yan-qiong Zhuang[a], and Zhong-fang Li[a]**

[a] College of Chemistry and Chemical Engineering, Shandong University of Science and Technology, Zibo 255000, Shandong, P. R. China.

*(Z-F Li) E-mail: zhfli@sdut.edu.cn; lizhongfangzb@126.com

ORCID *https://orcid.org/0000-0002-6480-2761*

Table of Contents

Table of Contents 2

S1 Experimental section 3

S1.1 Materials and Characterization. 3

S1.2 Synthesis of meso- 5,10,15,20-Tetra(4-cyanophenyl)porphyrin (TCNPorH_2) 3

S1.3 Synthesis of meso-5,10,15,20-Tetra(4-cyanophenyl)porphyrin Cobalt (TCNPorCo) 4

S1.4 Synthesis of 3D-G 4

S1.5 Preparation of the other sample 5

S2 Physical characterization 5

S3 Theoretical calculations 7

S4 Electrochemical Measurements 9

S5 Zn- Air Battery Assembly 12

S6 Supplementary Figures and Tables 14

S7 Reference 51

S1 Experimental section

S1.1 Materials and Characterization.

4-Cyanobenzaldehyde, Cobalt (II) acetate, 3,3'-diaminobenzidine (DAB), N, N-Dimethylformamide (DMF), N, N-Dimethylacetamide (DMAc), Acetic acid, Propionic acid, Nitrobenzene, Methanol, and potassium hydroxide (KOH) were purchased from Sinopharm Chemical Reagent Co., Ltd. Coal tar pitch (CTP) was purchased from a local chemical factory. $CaCO_3$ (20 nm) was purchased from Taiji Ring Nano Co. Ltd. Nafion was purchased from DuPont Company. Platinum-carbon (Pt/C) catalyst (20.0%) and RuO_2 were purchased from E-TEK Co. Ltd. and Anhui Senrise Technology Co. Ltd., respectively. Carbon cloth was purchased from CeTech Co. Ltd. 3,3'-diaminobenzidine (DAB) and Pyrrole were purchased from Energy Chemical Co. Ltd. All chemicals are AR grade, and no further purification is required for use.

S1.2 Synthesis of meso- 5,10,15,20-Tetra(4-cyanophenyl)porphyrin (TCNPorH$_2$)

The mixture of 80 mL of acetic acid, 80 mL of propionic acid, and 20 mL of nitrobenzene was heated to 150°C. Then, 4-cyanobenzaldehyde (26.2 g, 0.2 mol) was dissolved in the above solution and stirred for 15 minutes. After that, the mixture was obtained by dissolving freshly distilled pyrrole (10.1 g, 0.15 mol) in 30 mL of nitrobenzene, dropwisely adding to the reactor and mechanically stirring for 1 h[1, 2]. Finally, the obtained mixture was cooled. After filtration, the purple filter cake was

washed with methanol resulting in 20.2 g of crude product. The crude product was recrystallized three times in 100 mL of DMF to give 14.6 g of purple product powder and named meso-5,10,15,20-Tetra(4-cyanophenyl) porphyrin ($TCNPorH_2$), with a yield of 42.9%.

S1.3 Synthesis of meso-5,10,15,20-Tetra(4-cyanophenyl)porphyrin Cobalt (TCNPorCo)

$TCNPorH_2$ (0.714 g, 1 mmol), cobalt acetate tetrahydrate (0.249 g, 1.2 mmol), N, N'-dimethylformamide (DMF, 150 mL) were added to a 250 mL round bottom flask under a N_2. The obtained mixture was refluxed (≈145°C) under N_2 for 6 h. After the reaction, the dark red mixture was transferred to boiling deionized (DI) water (100 mL) and filtered while hot to remove excess cobalt ions three times. The lower layer was centrifuged and the precipitate was dried overnight in a vacuum oven at 60 °C to obtain TCNPorCo as dark purple solid powders. The red crystal powder was obtained by recrystallization of dichloromethane and n-hexane (V_{DCM}: V_{HEX}= 4:1) three times. (0.586 g, yield 75.9%).

S1.4 Synthesis of 3D-G

CTP (4 g) was dispersed in xylene (50 mL) under stirring at 100°C for 30 min. KOH (4 g) and $CaCO_3$ (8 g) (20 nm as template) were ground and dispersed into 50 mL of xylene. The mixture was stirred for 30 minutes and added to the above solution of CTP. The obtained mixture was evaporated to at 100 ° C under stirring until it was a paste. This paste was dried at 70 °C in an oven for 10 h. The gained brown precursor

was sufficiently ground and transferred into a porcelain boat, which was calcined for 2 h under a N_2 atmosphere at 900 ^{o}C. The black products were ground and washed with 10% HCl solution and deionized (DI) water four times. Finally, the powder was filtered and dried at 70 ^{o}C for 12 h in an oven, which was named **3D-G** (1 g, yield 25%)[3, 4].

S1.5 Preparation of the other sample

The series of samples were prepared according to the mass ratio of 3D-G and PBIPorCo (1:2, 1:1, and 2:1), all heat-treated at 600°C, and the samples were named PBIPorCo/3D-G-1-2, PBIPorCo/3D-G, PBIPorCo/3D-G-2-1. PBIPorCo/3D-G prepared under 500°C, 600°C, and 700°C were named as PBIPorCo/3D-G -500, PBIPorCo/3D-G, and PBIPorCo/3D-G-700, respectively. All ratio was 1:1 ($m_{PBIPorCo}$:$m_{3D\text{-}G}$).

TCNPorCo/3D-G was prepared using TCNPorCo with 3D-G as the precursor, following the preparation method of PBIPOrCo/3D-G.

S2 Physical characterization

The prepared materials were qualitatively analyzed by Ultraviolet–Visible spectra (UV–Vis) and UV–Vis diffuse reflectance absorption spectra (UV–Vis-DRS). A Nicolet 5700 transmission spectrophotometer was used to characterize the FT-IR spectra using the KBr pellet technique. Thermogravimetric (TG) analysis, carried out using STA-409 Netsch under a nitrogen environment with a flow rate of 30.0 mL min^{-}

^{1} and a heating rate of 10 °C min^{-1}, was used to investigate the material's thermal stability. The ^{1}H nuclear magnetic resonance (NMR) and ^{13}C solid-state nuclear magnetic were measured on Bruker ADVANCE III 400. On a PHI5300 ESCA spectrometer, the XPS (Perkin Elmer Instruments, Waltham, MA) analyzes the elemental makeup and valence states of the materials. The micromorphology of the samples was observed with SEM (FEI Sirion 200) and TEM (JEOL 2100). The XRD (D8 Advance, Bruker, German) was used to characterize the crystal structure of the samples with Cu-Ka radiation (k = 1.54178 Å) at a speed of 6° min^{-1}. A Raman spectrometer (HORIBA, Japan) with a wavelength of 532 nm was used to record the Raman spectra. Micromeritics ASAP 2020 instrument was employed to analyze the sample's BET area and pore size distribution. The inductively coupled plasma mass spectroscopy (ICP-MS) analysis was performed on an Agilent 7500 ce (Agilent Technologies, Tokyo, Japan).

The crystal structure of the as-prepared sample was characterized by powder X-ray diffraction (XRD) through an Ultima IV X-ray diffractometer with $Cu\ K_{\alpha}$ radiation (λ = 0.15406 nm). Bragg's law formula as shown:

$$2dsin\theta = n\lambda \qquad [1]$$

d is the plane distance (nm), and θ is angle of incidence (2 θ , °), n =1.

The specific surface area was measured by the Brunauer-Emmett-Teller (BET) method and the Barrett–Joyner–Halenda type that adsorption with N_2 at -196 °C, which was conducted on a Micromeritics ASAP 2010 instrument. The surface morphology of catalysts was tested using field-emission scanning electron microscopic (FE-SEM, Apreo), and their microstructures were investigated by high-resolution transmission electron microscopic (HR-TEM, FEI-Tecnai TF20, 200 kV). X-ray photoelectron spectroscopy (XPS, PHI 5000 Versaprobe III) was used to analyze the surface composition and valent state of samples[5]. The Raman spectrometer (HORIBA, Japan) records the Raman spectrum with a wavelength of 532 nm.

S3 Theoretical calculations

The calculation uses the basis group of 6-31+G(d) and the generalized function of B3LYP-D3BJ containing the dispersion effect. Gaussian16 (G16 A.01) software was used to optimize the monomeric cobalt phthalocyanine (TCNPorCo) and conjugated PBIPorCo ground state, to calculate the HUMO and LUMO orbitals of the molecules, the electrostatic potential (ESP) and the adsorption energy for oxygen molecule. Multiwfn version 3.8 (dev) [35] was used to analyze the wave function files of the Gaussian output and visualize the results of the analysis by VMD-Visual Molecular Dynamics

The first-principles DFT calculations were investigated within the generalized gradient approximation (GGA) through the Vienna abinitio simulation package

(VASP). The molecular models featuring periodicity were built in both the x and y directions, and the vacuum layer's depth exceeded 5 Å to avoid any self-interactions. The calculations were conducted by the GGA using the Perdew-Burke-Ernzerhof (PBE) formulation. The projector augmented wave (PAW) potential to describe the interactions and the Kohn-Sham one-electron valence states were expanded based on plane waves with a cutoff energy of 475.35 eV. The Hellmann-Feynman forces convergence criterion was established at less than 0.05 eV $Å^{-1}$, and a k-point grid of 1×1×3 was employed for optimizing all molecular models[6, 7].

Additionally, the electrostatic potential and Electron density difference analysis were conducted during single-point energy calculations. The electronic energy is considered to be self-coincidental when the energy gap is less than 10^{-5} eV. The k-point parameter and cutoff energy are consistent with the process of structure optimization[8].

ORR steps:

$$* + O_2(g) + H_2O(l) + e^- \rightarrow *OOH + OH^- \quad (1)$$

$$*OOH + e^- \rightarrow *O + OH^- \quad (2)$$

$$* O + H_2O(l) + e^- \rightarrow * OH + OH^- \quad (3)$$

$$* OH + e^- \rightarrow * + OH^- \quad (4)$$

The change in Gibbs free energy (ΔG) of ORR/OER intermediates was calculated using

$\Delta G = \Delta E + \Delta \mathrm{ZPE} + T\Delta S - \mathrm{e}U$ [9]

Where ΔE is the energy change at each step of the reaction, ΔZPE is the zero point energy. ΔZPE is the change in ZPE calculated from the vibrational frequencies and ΔS is the change in the entropy referring to thermodynamics databases. T is the temperature (298.15 K), and ΔS is the change in entropy. The ΔZPE was calculated by using the vibrational frequency of intermediates. The corrected values of the free energy are calculated by VASPKIT, a plug-in for VASP. The electrode potential is adopted with respect to the reversible hydrogen electrode, which makes the standard electrochemical potential of the electron involved in the reaction (Ge) equal to -eU, and the standard electrochemical potential of the proton (G_{H+}) equal to that of the hydrogen atom in gaseous H_2 ($1/2G_{H2}$). Considering that the triplet state of the O_2 molecule is poorly described in the current DFT scheme, the free energy of the O_2 molecule was derived according to $G_{O2} = 2G_{H2O} - 2G_{H2} + 4.92$.

S4 Electrochemical Measurements

The work was measured by CHI 760e electrochemical workstation (CH Instruments Inc.) at 30 °C. The ORR tests which include Cyclic voltammetry and Linear sweep voltammetry (LSV) were obtained by a three-electrode cell, which include glass carbon as worker, counter carbon, and Ag/AgCl reference electrode. The potential of the reference electrode was calibrated to the reversible hydrogen electrode (RHE): E_{RHE}= ($E_{Ag/AgCl}$=0.197 + 0.05916×pH) V. As work electrode, the glassy carbon rotating-disk electrode (diameter 5 mm; surface area 0.196 cm^2) was extremely polished with alumina slurry to achieve a mirror-like surface. The electrode surface was washed with ethanol and deionized water and then dried at room temperature[10].

For the RRDE measurements, the disk electrode was scanned cathodically at a rate of 10 mV s^{-1}, and the ring potential was kept at 1.500 V *vs*. RHE. The ring-disk electrode composed of a glassy carbon disk (d=5.61 mm) and a Pt ring (6.25 mm inner diameter and 7.92 mm outer diameter) served as the working electrode with 200 μg/cm^2 catalyst loading[4].

To prepare the catalyst ink, the catalyst sample was dispersed in 1 mL of ethanol and dropped into 10 μL 5 wt% Nafion solution. After 30 minutes of ultrasound, a uniform ink solution was formed. During the test, apply 10 μL of ink evenly to the

surface of the electrode and dry it in air. Before the test, 0.1 mol/L KOH electrolyte was injected into N_2 for 30 minutes, and then the CV curve of the catalyst was obtained in the atmosphere of saturated N_2[11]. After the CV test in an atmosphere of saturated N_2, the O_2 was imported into 1 mol/L KOH for 30 minutes. The ORR electrochemical measurements of LSV and CV were carried out at 50 mV/s and 10 mV/s in 0.1 mol/L KOH, and the curves of LSV were obtained at the potential range of -0.8 V-0.2 V, then the electron transfer number was calculated by the Koutecky–Levich (K-L) equations, which further elucidated the ORR kinetics[12].

$$\frac{1}{J}=\frac{1}{J_K}+\frac{1}{J_L}=\frac{1}{J_K}+\frac{1}{B\omega^{\frac{1}{2}}} \qquad [2]$$

$$B=0.62nFC_0{D_0}^{2/3}v^{-1/6} \qquad [3]$$

$$J_K=nFkC_0 \qquad [4]$$

The J [mA·cm^{-2}] is the current density. J_L and J_K are diffusion-limiting current density and kinetic current density. ω is the rotation rate of the disk electrode (rad/s), The value of *F,* which means Faraday constant, is 96,485C·mol $^{-1}$. *n* is the electron transfer number in ORR; C_0 represent the volume of O_2 which was dissolved in the electrolyte solution, and *k* is the electron transfer constant. D_0 is the diffusion coefficient of O_2 in the electrolyte (0.1 mol/L KOH), and *v* is the kinetic viscosity of the electrolyte.

RRDE measurements were carried out to determine four-electron selectivity, and the disk electrode was scanned at a rate of 10 mV s^{-1}.The n and hydrogen peroxide yield (H_2O_2%) were calculated from RRDE measurement according to the following equations:

$$n = \frac{4I_D}{I_D + \frac{I_R}{N}} \qquad [5]$$

$$H_2O_2\% = \frac{\frac{2I_D}{N}}{I_D + \frac{I_R}{N}} \times 100\% \qquad [6]$$

I_D and I_R represented the disk current and ring current, respectively, and N was the ring collecting efficiency (37 %).

S5 Zn- Air Battery Assembly

The prepared catalyst ink is uniformly sprayed on the carbon cloth hydrophilic layer, the catalyst spraying ratio is 500 ug/cm^2. Nickel foam was used as the collector layer of the air cathode and a polished zinc plate was used as anode. The electrolyte was selected as 6 mol/L KOH and 0.2 mol/L Zn^{2+} solution[13].

F-ZABs are prepared using polyvinyl alcohol (PVA) as an electrolyte. PVA was prepared as follows:

Firstly, 1 g of PVA was added to 10 mL of deionized water and heating it at 95 ^{o}C for 30 min. After the mixture became uniform and viscous, 30 mL of 6 mol/L KOH and 0.2 mol/L Zn^{2+} electrolyte was added into PVA gel, and then heated for 15 min, stirring continuously, PVA solidified to get a solid gel. The PVA gel was extruded from two glass plates to form a sheet gel, which was then frozen for 10 h to form a solid electrolyte[14].

The charge-discharge curve and specific capacity of ZABs were tested by the electronic load instrument (PV8500). The impedance of ZABs was tested using an electrochemical workstation (Ivium). ZABs durability test using a battery test system (NEWARE CT-1008).

S6 Supplementary Figures and Tables

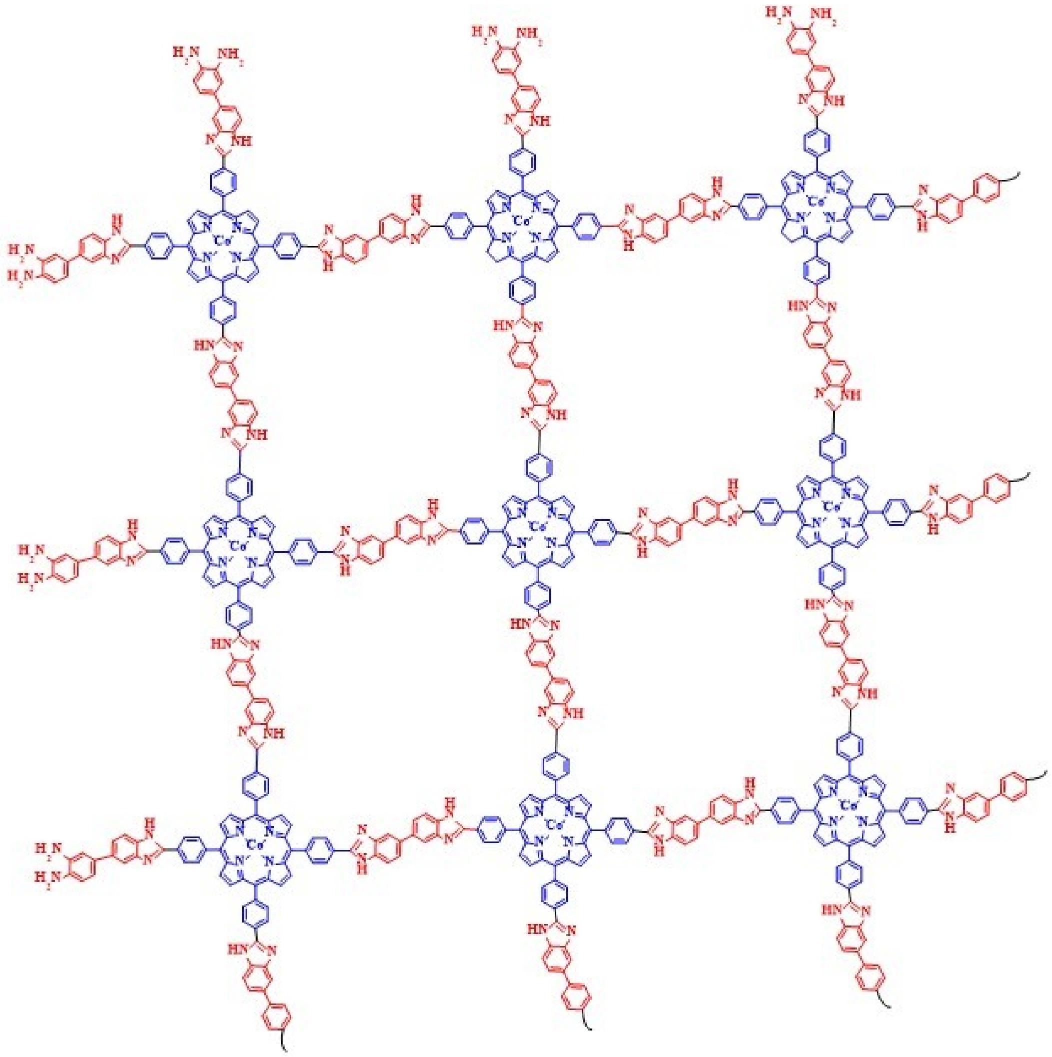

Figure S1. The structural formula of PBIPorCo.

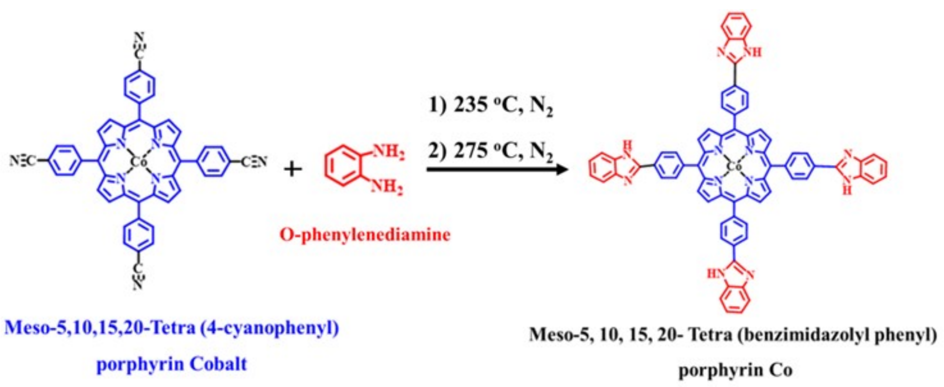

Figure S2. Synthesis of Meso-5,10,15,20- Tetra (benzimidazolyl phenyl) porphyrin Co(II) (TBIPorCo)

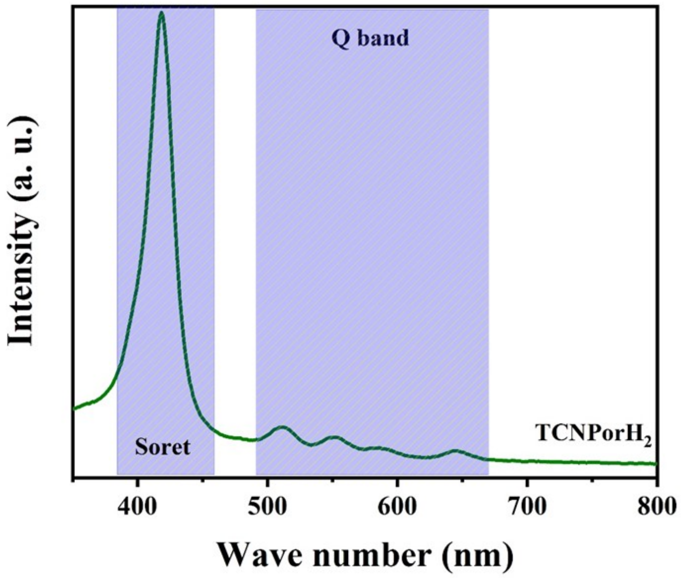

Figure S3. UV-Vis of $TCNPorH_2$

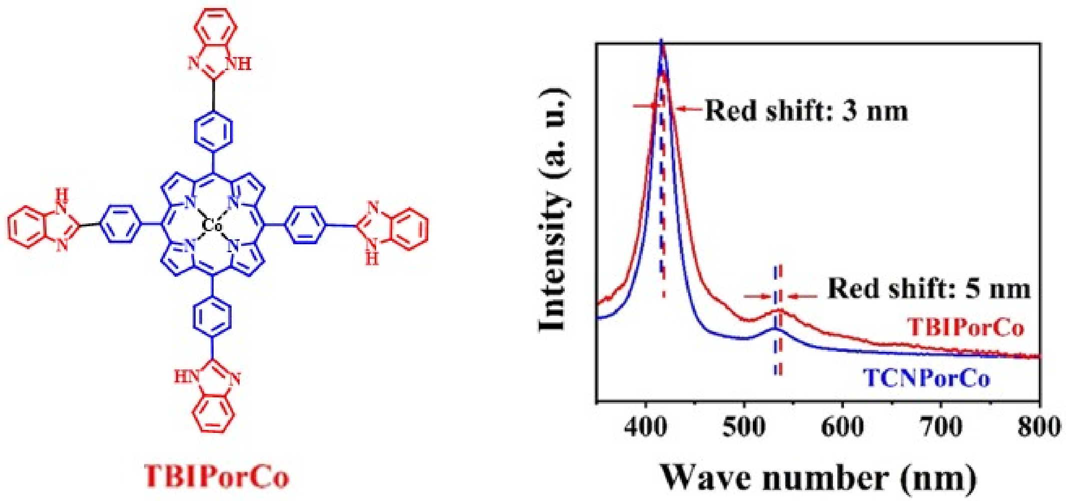

Figure S4. The UV-Vis of TBIPorCo

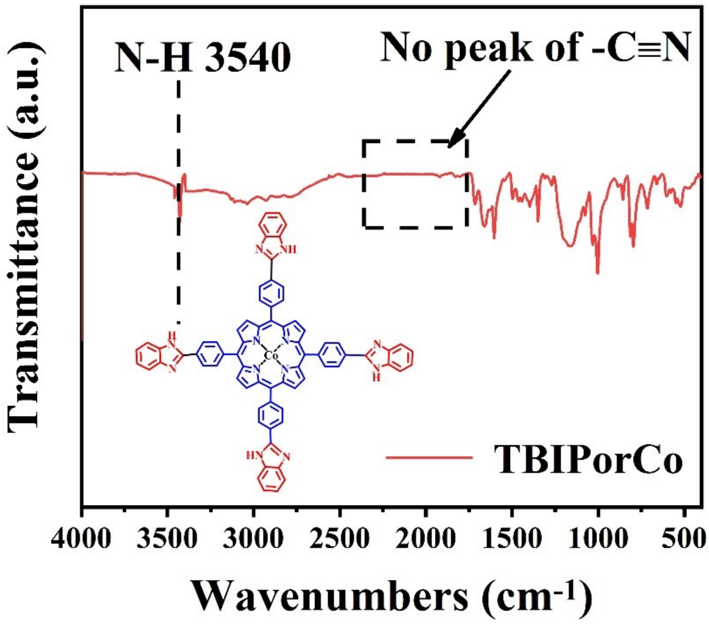

Figure S5. The FT-IR of TBIPorCo

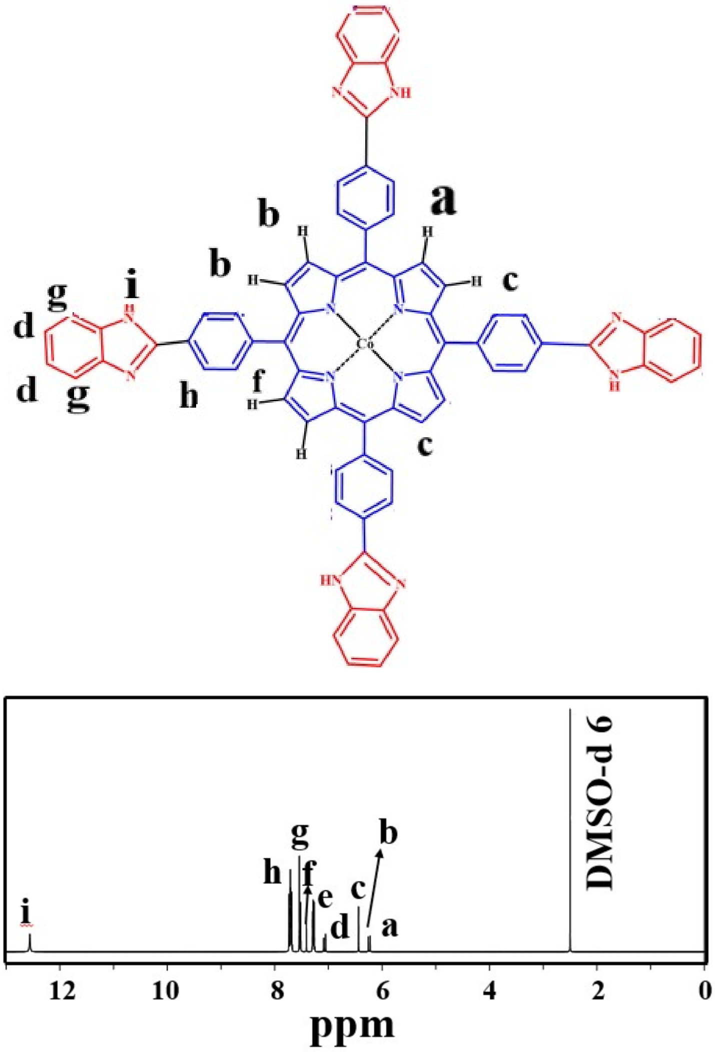

Figure S6. The ^{1}HNMR of TBIPorCo

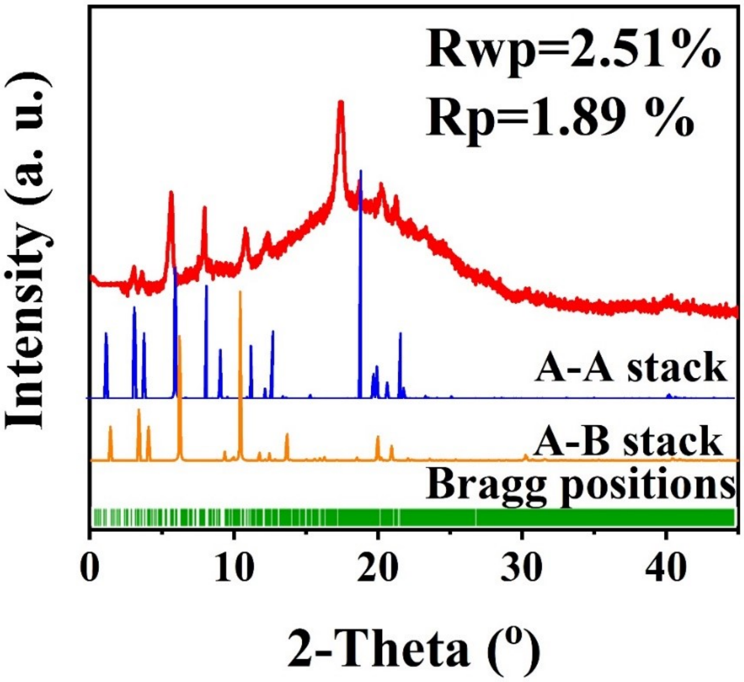

Figure S7. The PXRD of PBIPorCo

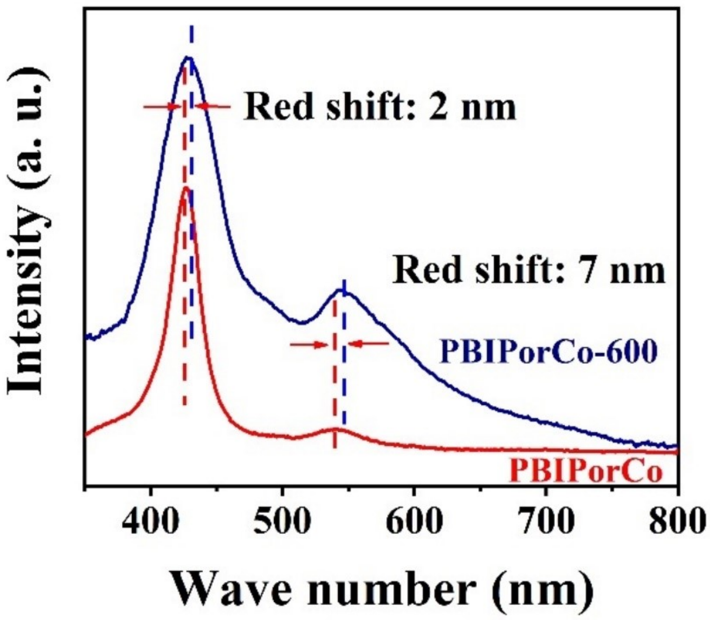

Figure S8. The UV-Vis of PBIPorCo-600 and PBIPorCo

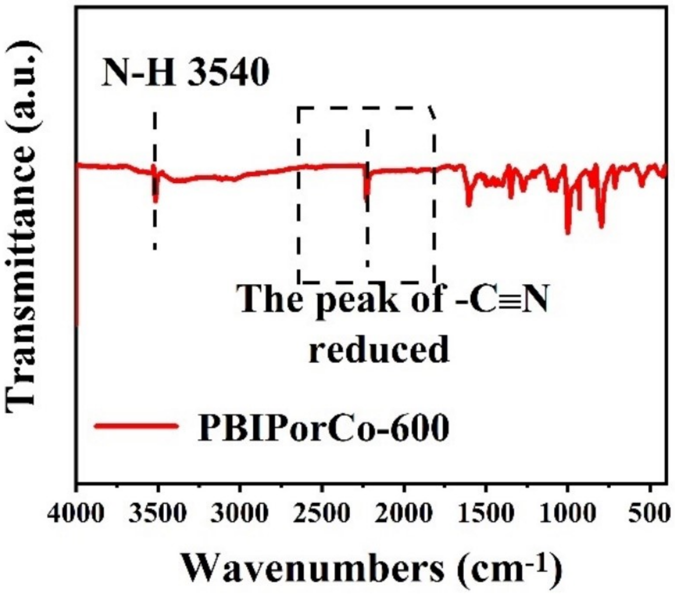

Figure S9. The FT-IR of PBIPorCo-600

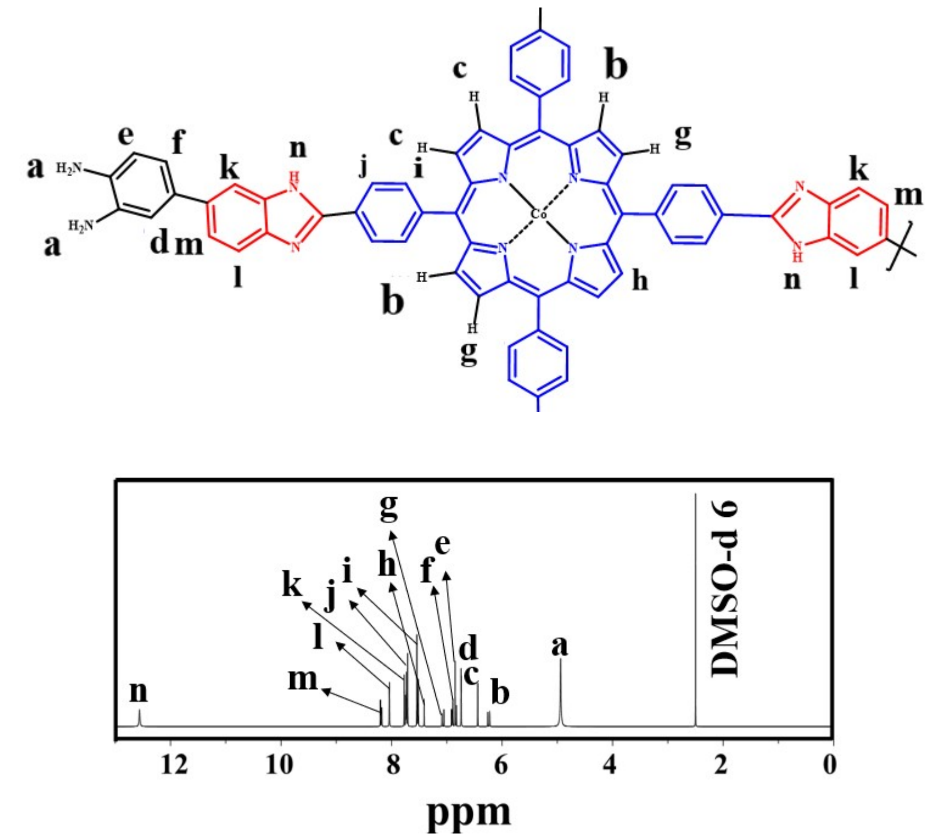

Figure S10. The ^{1}HNMR of PBIPorCo-600

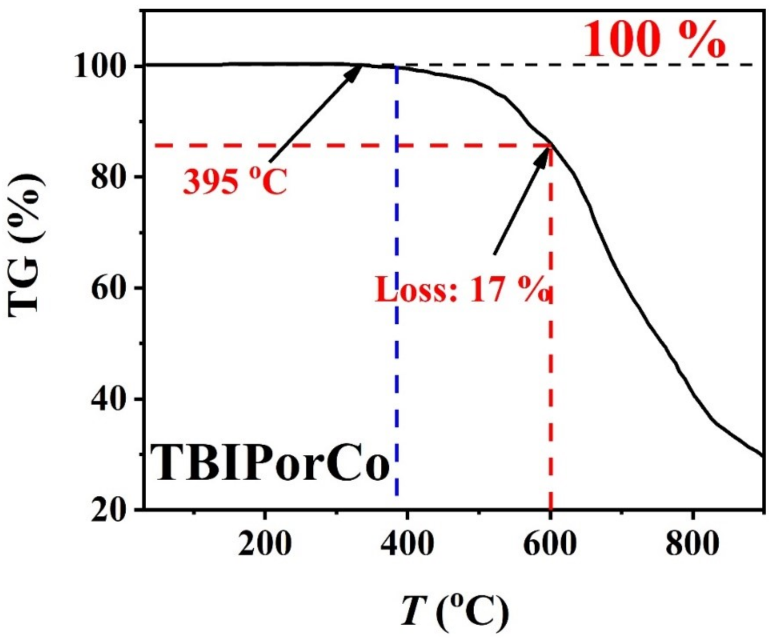

Figure S11. The TG of TBIPorCo

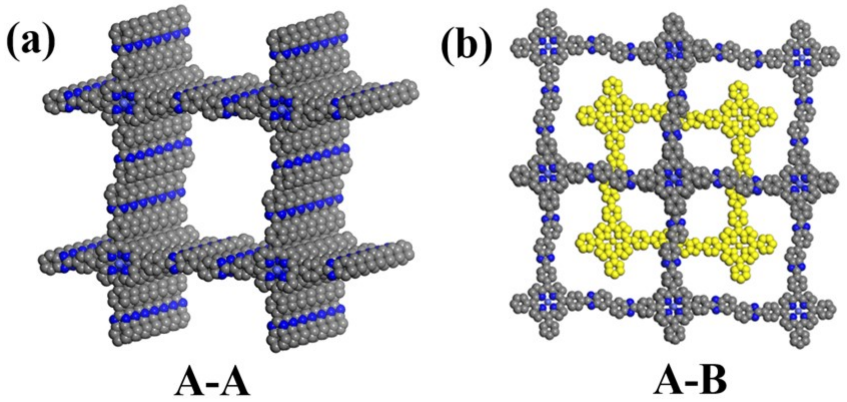

Figure S12. (a) A-A and (b) A-B stacking style model of PBIPorCo

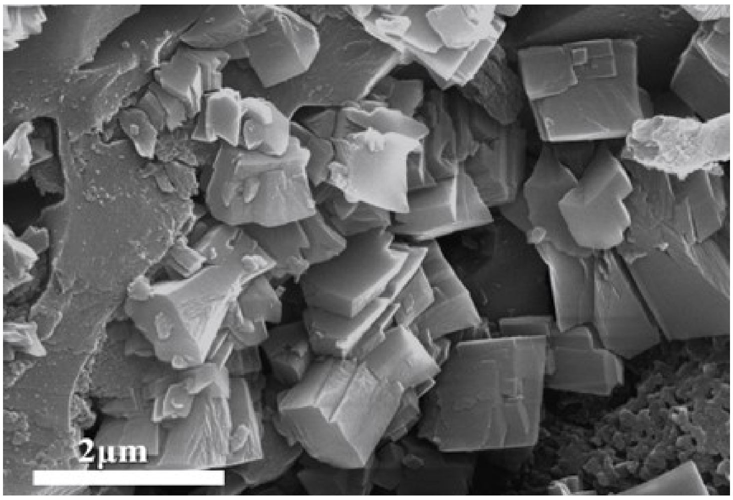

Figure S13. The SEM of PBIPorCo

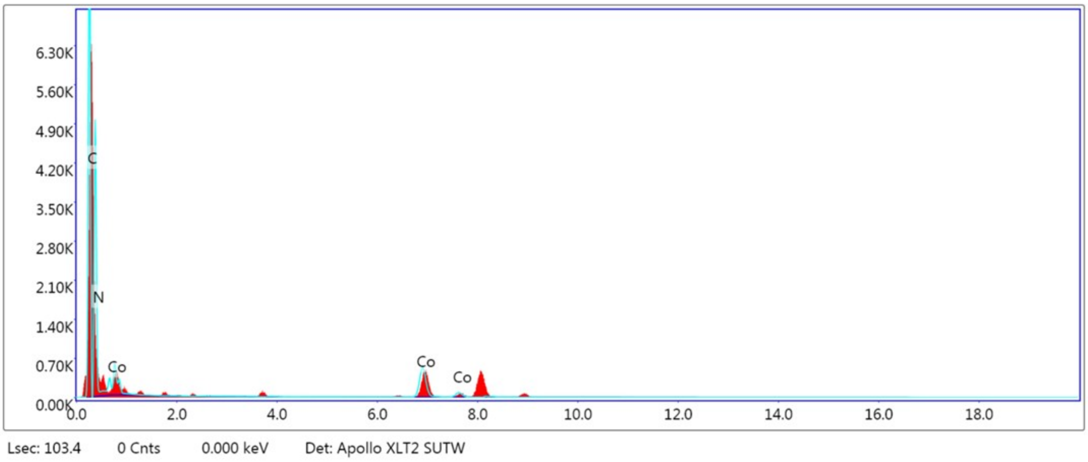

Element	Weight (%)	Atom (%)
C	42.89	50.2
N	42.09	42.24
Co	15.02	7.56

Figure S14. Corresponding EDX spectrum of PBIPorCo

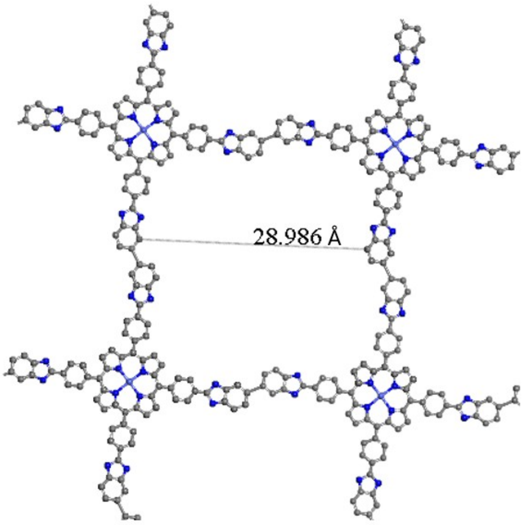

Figure S15. The theoretical pore sizes for the COF

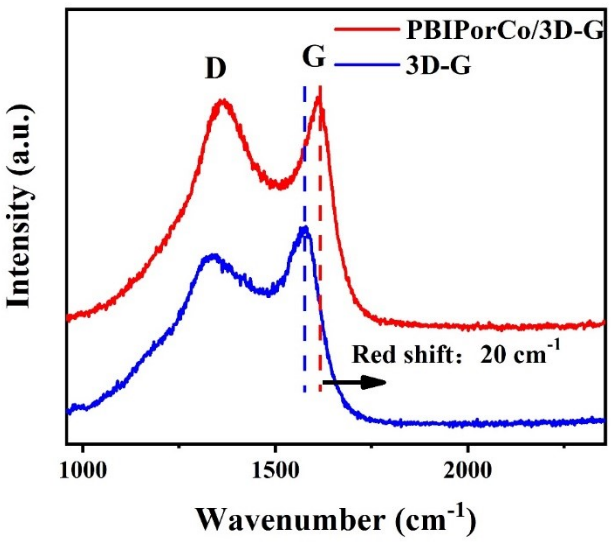

Figure S16. The G-peak region of the Raman spectra

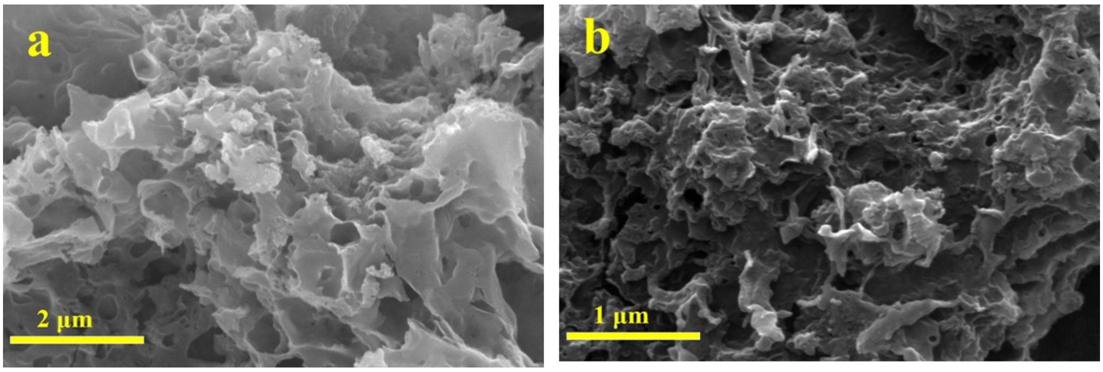

Figure S17. SEM of PBIPorCo/3D-G

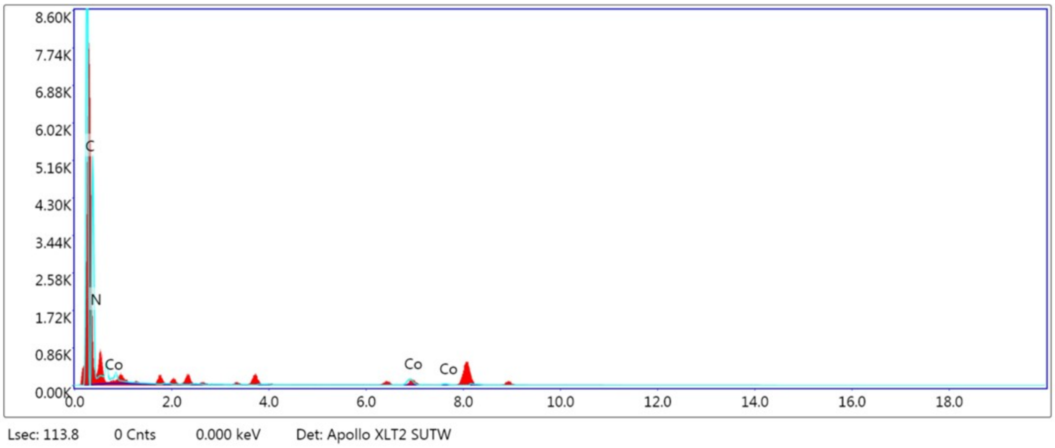

Element	Weight (%)	Atom (%)
C	57.46	62.35
N	39.80	37.04
Co	2.74	4.56

Figure S18. Corresponding EDX spectrum of PBIPorCo/3D-G

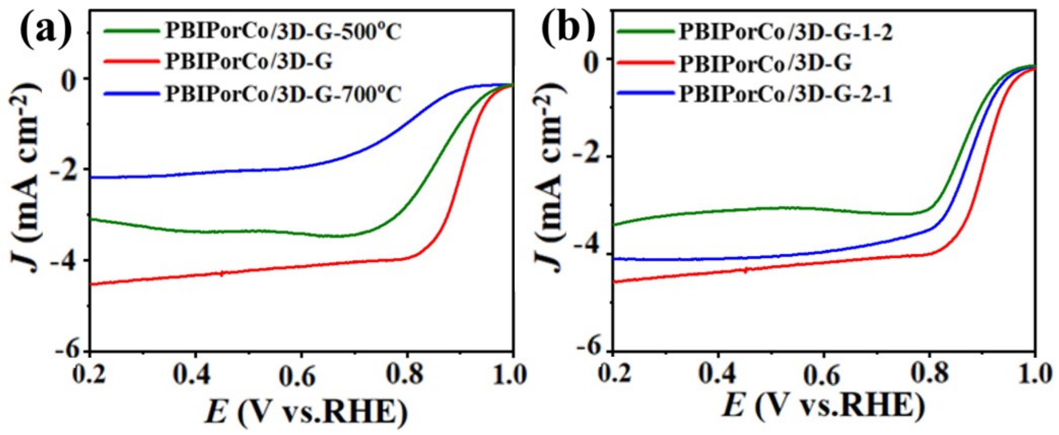

Figure S19. The ORR performance of PBIPorCo/3D-G-500°C, PBIPorCo/3D-G-700°C, PBIPorCo/3D-G, PBIPorCo/3D-G-2-1, and PBIPorCo/3D-G-1-2

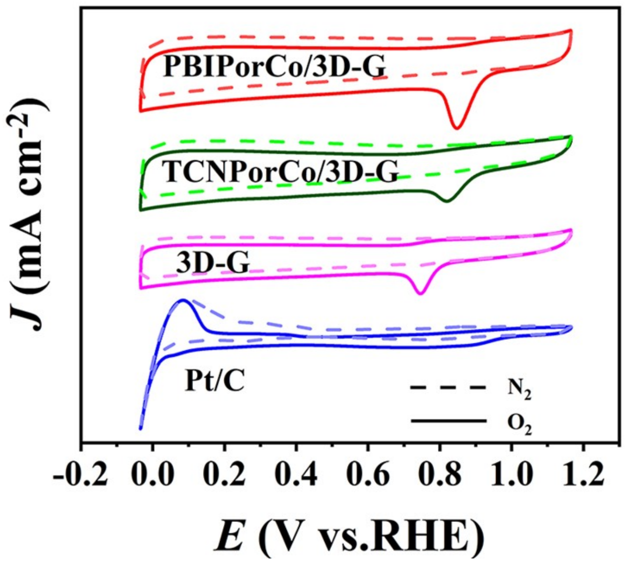

Figure S20. The ORR test of PBIPorCo/3D-G, TCNPorCo/3D-G, 3D-G, and 20wt% Pt/C in 0.1 mol L^{-1} KOH: (a) CV of in O_2 and N_2 saturated KOH solution

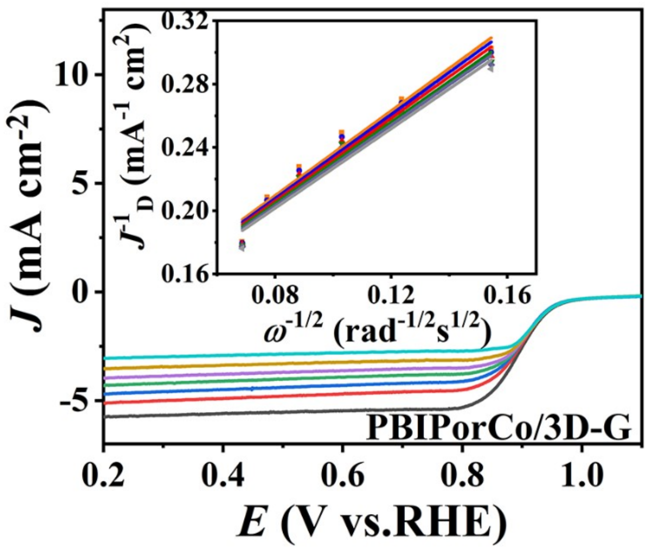

Figure S21. LSV curves of PBIPorCo/3D-G with the rotation speed from 400 to 2500 rpm and K-L curves

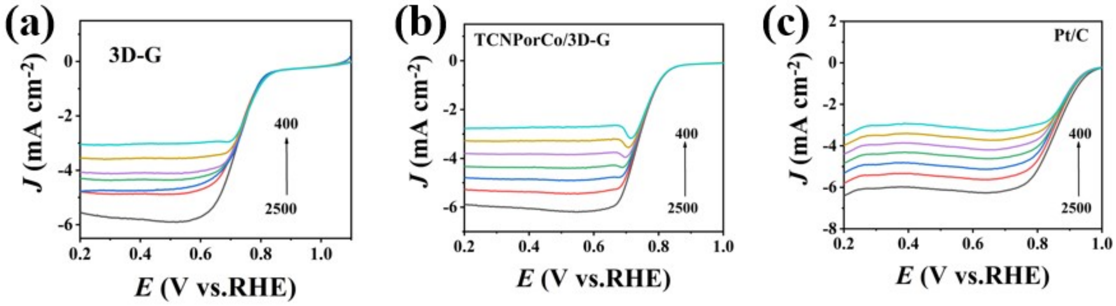

Figure S22. LSV curves of (a) 3D-G; (b) TCNPorCo/3D-G; (c) Pt/C with the rotation speed from 400 to 2500 rpm

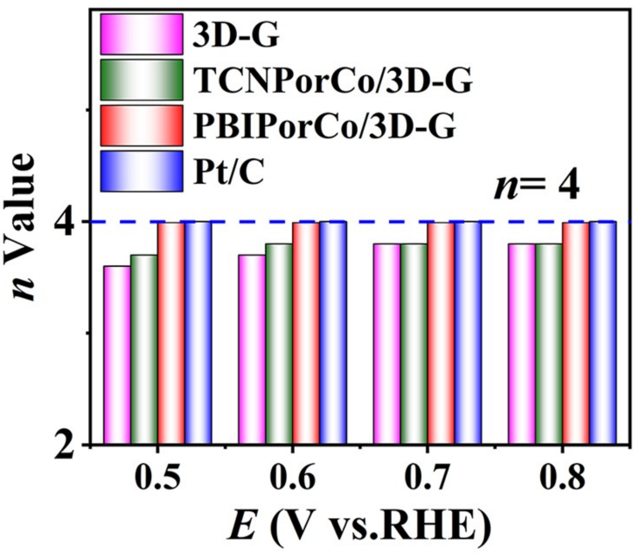

Figure S23. The number of electrons transferred from K-L curves

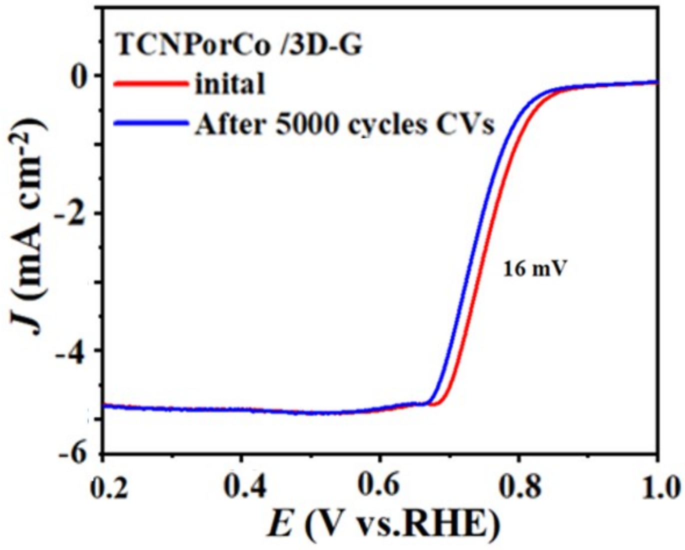

Figure S24. The LSV curves of ORR for TCNPorCo/3D-G before and after 5000 cycles

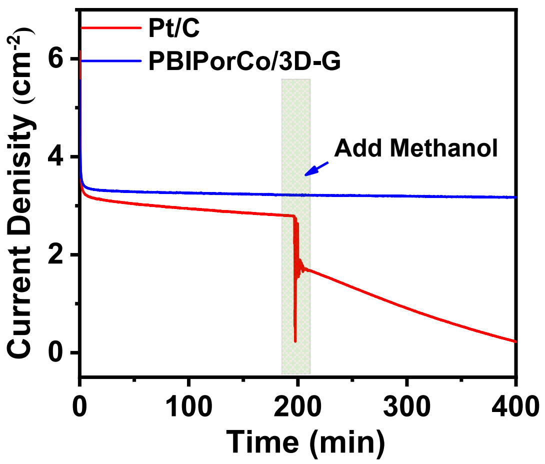

Figure S25. The methanol resistance test

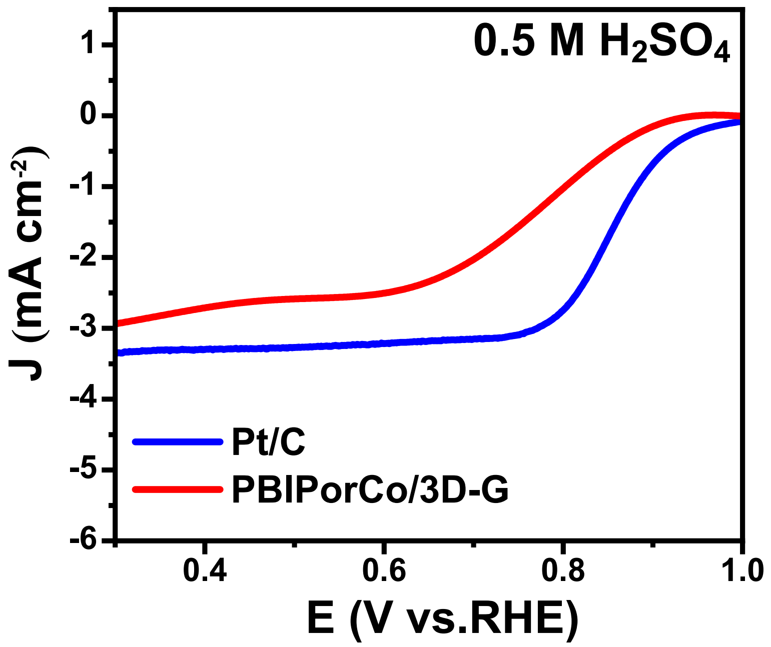

Figure S26. The catalyst performance of ORR in 0.5 M H_2SO_4

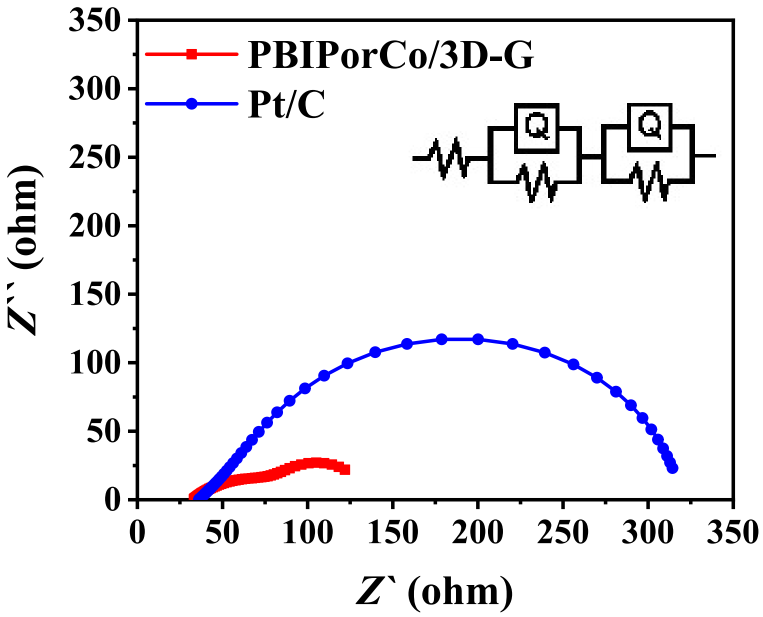

Figure S27. The ORR electrochemical impedance spectroscopy (EIS) of PBIPorCo/3D-G and compare with Pt/C

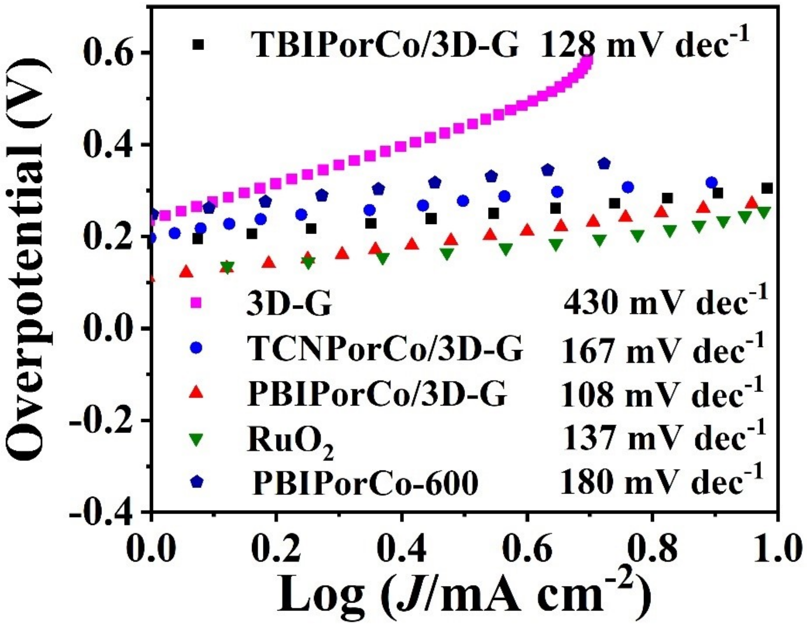

Figure S28. The OER Tafel curve in 1 mol L^{-1} KOH of PBIPorCo/3D-G, TCNPorCo/3D-G, RuO_2, PBIPorCo-600, TBIPorCo/3D-G, and 3D-G

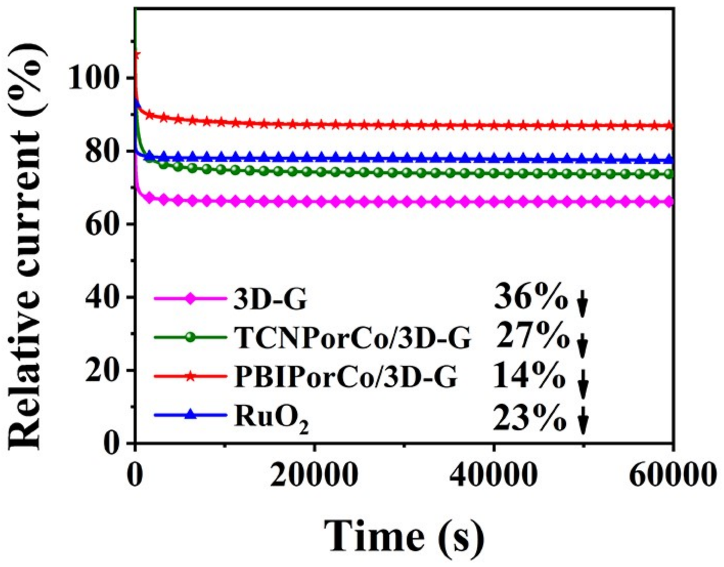

Figure S29. The OER test of PBIPorCo/3D-G, TCNPorCo/3D-G, RuO_2, and 3D-G in 1 mol L^{-1} KOH: *i-t* curve

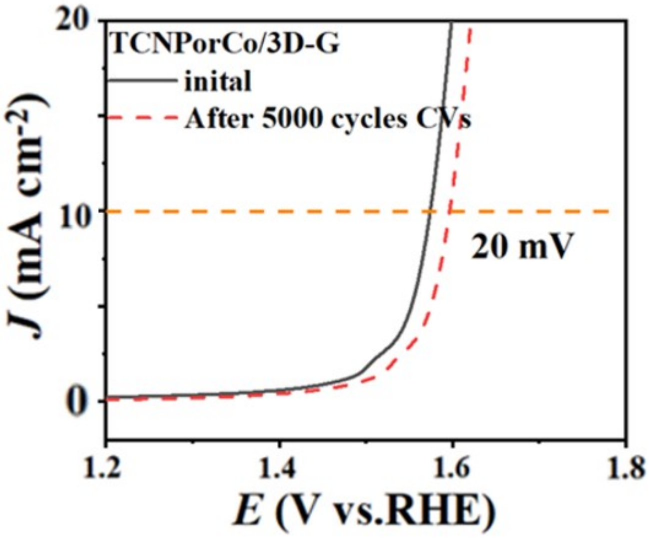

Figure S30. The ADT curve of TCNPorCo/3D-G

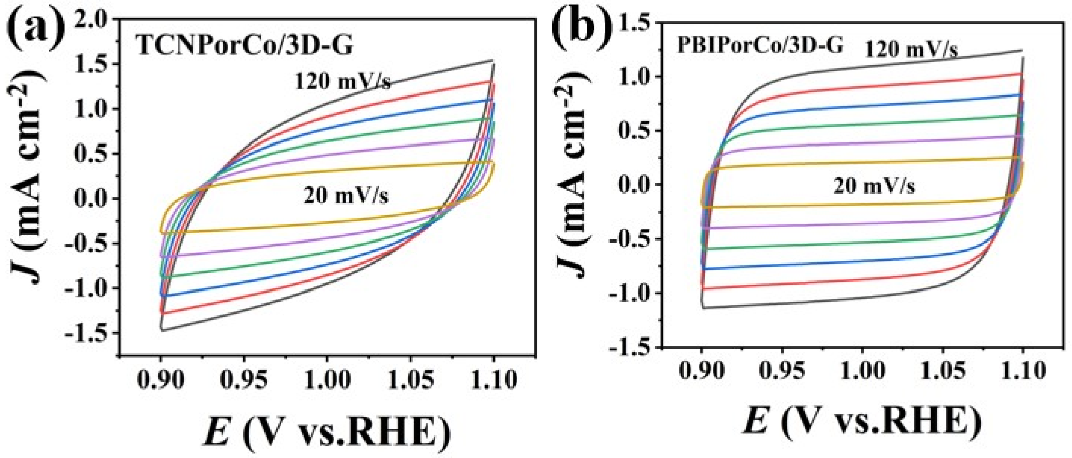

Figure S31. Cyclic voltammograms of (a) TCNPorCo/3D-G; (b) PBIPorCo/3D-G

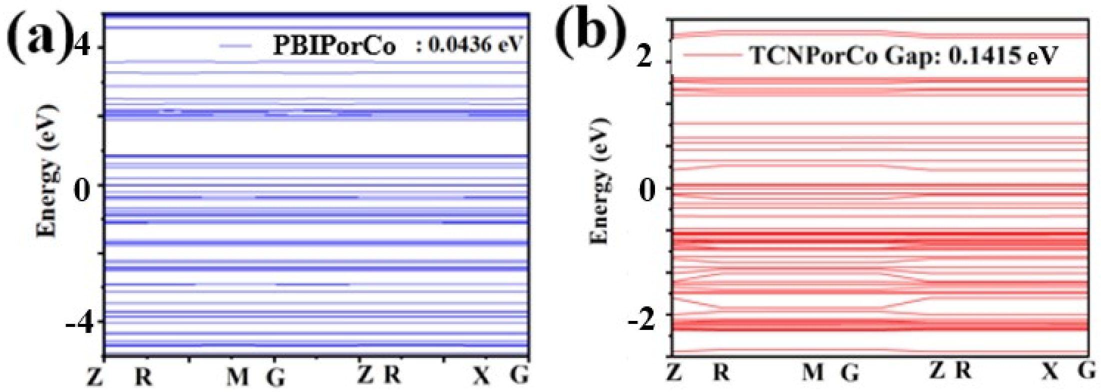

Figure S32. The band structure of (a)PBIPorCo and (b)TCNPorCo

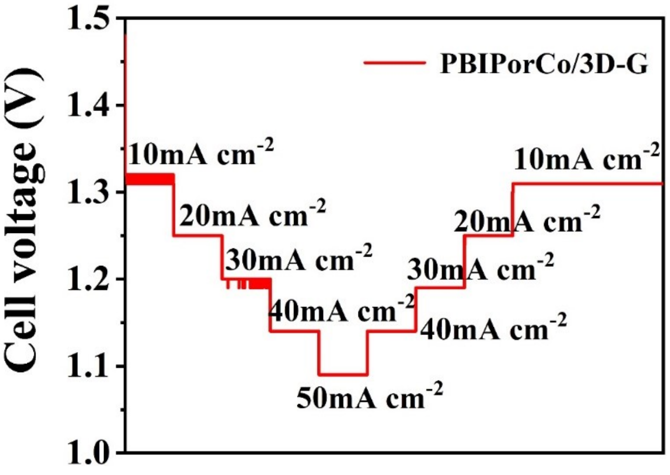

Figure S33. Step-discharged at different currents

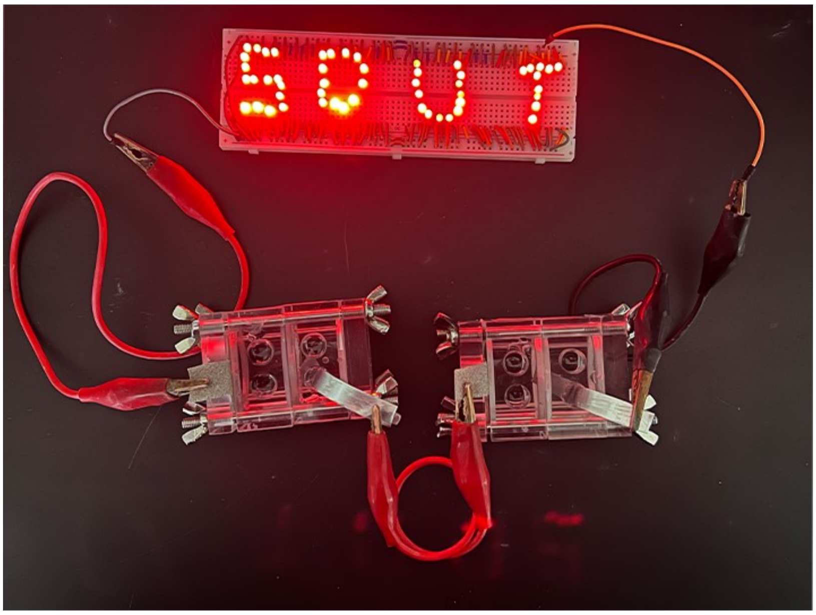

Figure S34. The emitting diode (~3.0 V) is powered by two liquid Zn-air batteries in series

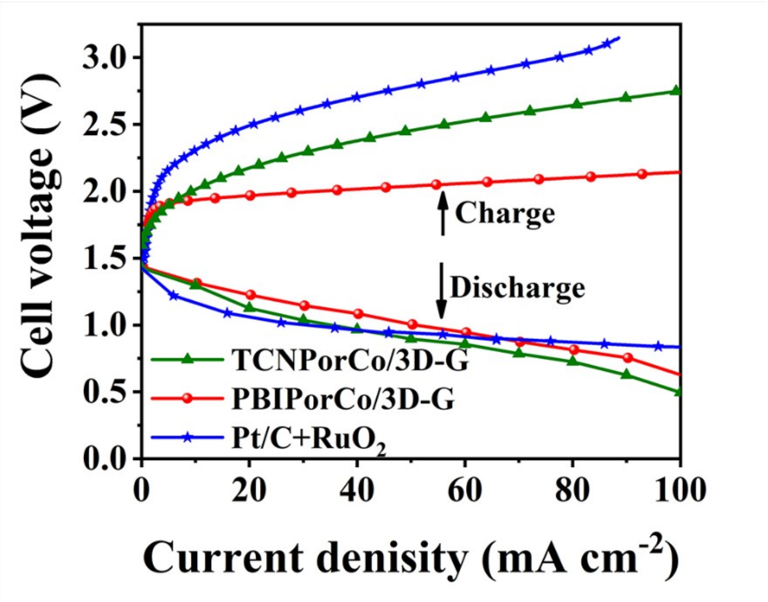

Figure S35. Charging-discharging polarization curves

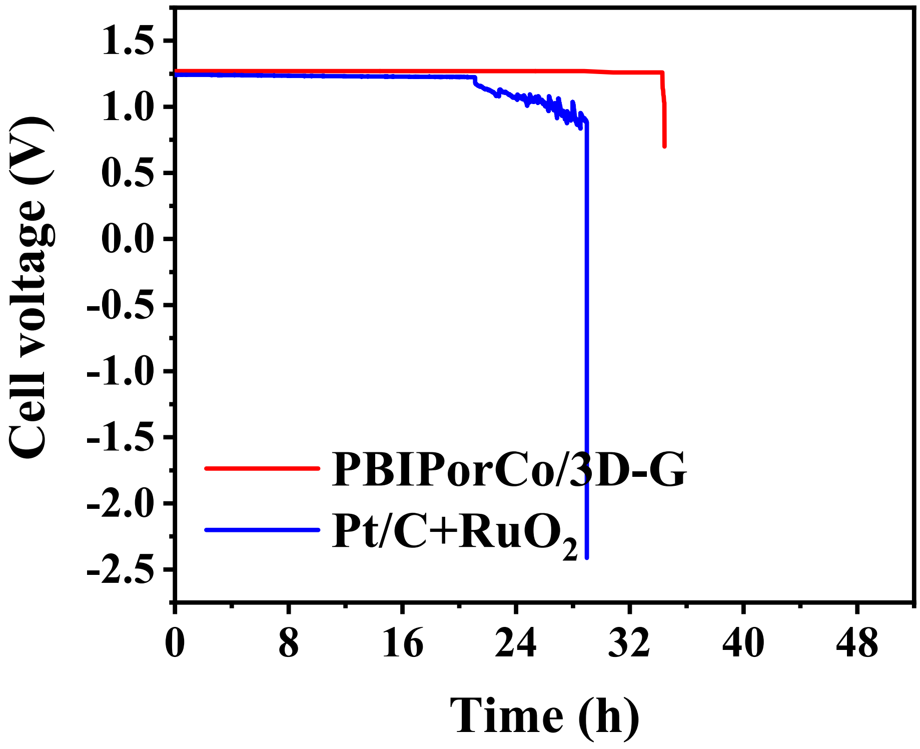

Figure 36. The specific discharging capacities of F-ZABs

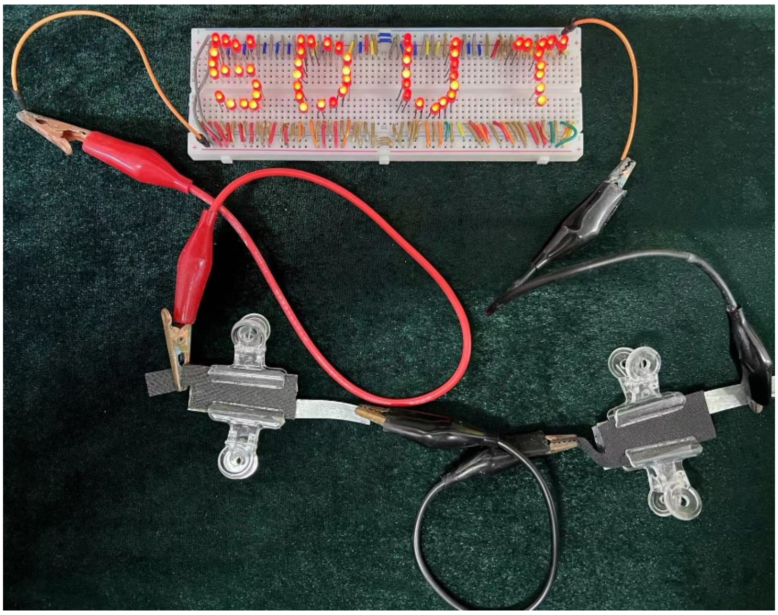

Figure S37. The LED lights were lit by F-ZABs

Table S1. The inductively coupled plasma mass spectroscopy (ICP-MS) for Co element of PBIPorCo and PBIPorCo/3D-G

Sample	Element	Test solution concentration (mg L^{-1})	Sample element content wt (%)
**PBIPorCo**	**Co**	**4.67**	**3.74**
**PBIPorCo/3D-G**	Co	2.04	1.63

Table S2. The Atomic % of PBIPorCo and PBIPorCo/3D-G

Sample	Co%	N%	C%
**PBIPorCo**	4.4	22.5	73.1
**PBIPorCo/3D-G**	2.6	14.7	82.7

Table S3. The Atomic % of PBIPorCo/3D-G-5000

Sample	Co%	N%	C%
**PBIPorCo/3D-G**	2.4	14.4	83.2

Table S4 The comparison of the other ZABs performance

Sample	Power density (mA cm^{-2})	Specific capacity (mA h g_{Zn}^{-1})	Reference
PBIPorCo/3D-G	356.8	817.2	**This work**
CoSA@NC	116.7	730.1	J. Colloid Interface Sci., 670 (2024) 103-113.
COFs-FeNi LDH	197	798	Adv. Mater., 35 (2023) 2210550
CC-3 (COF/CNT)	85	712	ACS Nano, 15 (2021) 3309-3319
CoFe-FeNC	120.8	767.5	Applied Catalysis B: Environment and Energy, 359 (2024) 124485
$NiCo_2S_4$@NiFe LDH/N-rGO	188	732	Energy Environ. Sci., (2025). 10.1039/d4ee03240c
Co-O-ZIF/PANI	123.1	781.1	J. Energy Chem., 68 (2022) 78-86.
*pf*SAC-Fe-0.2	123.43	732	Sci. Adv., 5 (2019) eaaw2322.
CoNi-MOF/rGO	97	711	ACS Appl. Mater. Interfaces, 11 (2019) 15662-15669.
Co-POC	78	-	Adv. Mater., 31 (2019). 1900592

S7 Reference

[1] Z. Li, J. Yang, G. Xu, S. Wang, *J. Power Sources* **2013**, 242, 157.
[2] S.-W. Ke, W. Li, Y. Gu, J. Su, Y. Liu, S. Yuan, J.-L. Zuo, J. Ma, P. He, *Science Advance* **2023**, 9, eadf2398.
[3] S. Wang, P. Sun, N. Li, J. Wang, L. Zhang, W. Duan, Z. Li, *J. Power Sources* **2023**, 556, 232471.
[4] Y. Liu, Z. Li, C. Sun, S. Wang, L. Wang, X. Niu, P. Sun, S. Zhang, *Sustainable Energy Fuels* **2021**, 5, 5216.
[5] Y. Yang, R. G. Agarwal, P. Hutchison, R. Rizo, A. V. Soudackov, X. Lu, E. Herrero, J. M. Feliu, S. Hammes-Schiffer, J. M. Mayer, H. D. Abruña, *Nat. Chem.* **2023**, 15, 271.
[6] C. E. Beall, E. Fabbri, T. J. Schmidt, *ACS Catal.* **2021**, 11, 3094.
[7] S. Wang, Z. Li, W. Duan, P. Sun, J. Wang, Q. Liu, L. Zhang, Y. Zhuang, *J. Energy Chem.* **2023**, 86, 41.
[8] F. Cheng, J. Shen, B. Peng, Y. Pan, Z. Tao, J. Chen, *Nat. Chem.* **2011**, 3, 79.
[9] V. Viswanathan, H. A. Hansen, J. Rossmeisl, J. K. Norskov, *J Phys Chem Lett* **2012**, 3, 2948.
[10] Q. Zhang, S. Dong, P. Shao, Y. Zhu, Z. Mu, D. Sheng, T. Zhang, X. Jiang, R. Shao, Z. Ren, J. Xie, X. Feng, B. Wang, *Science* **2022**, 378, 181.
[11] T. Stracensky, L. Jiao, Q. Sun, E. Liu, F. Yang, S. Zhong, D. A. Cullen, D. J. Myers, A. J. Kropf, Q. Jia, S. Mukerjee, H. Xu, *ACS Catal.* **2023**, 13, 14782.
[12] S. Liu, Z. Wang, S. Zhou, F. Yu, M. Yu, C. Y. Chiang, W. Zhou, J. Zhao, J. Qiu, *Adv. Mater.* **2017**, 29, 1700874.
[13] S. Liu, B. Zhang, Y. Cao, H. Wang, Y. Zhang, S. Zhang, Y. Li, H. Gong, S. Liu, Z. Yang, J. Sun, *ACS Energy Lett.* **2022**, 8, 159.
[14] Z. Niu, Z. Lu, Z. Qiao, M. Xing, L. Han, S. Wang, D. Cao, *ACS Catal.* **2023**, 13, 7122.
